# Beauty Is in the AI of the Beholder: Are We Ready for the Clinical Integration of Artificial Intelligence in Radiography? An Exploratory Analysis of Perceived AI Knowledge, Skills, Confidence, and Education Perspectives of UK Radiographers

**DOI:** 10.3389/fdgth.2021.739327

**Published:** 2021-11-11

**Authors:** Clare Rainey, Tracy O'Regan, Jacqueline Matthew, Emily Skelton, Nick Woznitza, Kwun-Ye Chu, Spencer Goodman, Jonathan McConnell, Ciara Hughes, Raymond Bond, Sonyia McFadden, Christina Malamateniou

**Affiliations:** ^1^Faculty of Life and Health Sciences, School of Health Sciences, Ulster University, Newtownabbey, United Kingdom; ^2^The Society and College of Radiographers, London, United Kingdom; ^3^School of Biomedical Engineering and Imaging Sciences, King's College London, St Thomas' Hospital, London, United Kingdom; ^4^Department of Radiography, Division of Midwifery and Radiography, School of Health Sciences, University of London, London, United Kingdom; ^5^University College London Hospitals, London, United Kingdom; ^6^School of Allied and Public Health Professions, Canterbury Christ Church University, Canterbury, United Kingdom; ^7^Department of Oncology, Oxford Institute for Radiation Oncology, University of Oxford, Oxford, United Kingdom; ^8^Radiotherapy Department, Churchill Hospital, Oxford University Hospitals NHS FT, Oxford, United Kingdom; ^9^NHS Scotland, Greater Glasgow and Clyde, Glasgow, United Kingdom; ^10^Faculty of Computing, Engineering and the Built Environment, School of Computing, Ulster University, Newtownabbey, United Kingdom

**Keywords:** artificial intelligence, AI, radiography, education, workforce training, digital health, radiotherapy, adoption

## Abstract

**Introduction:** The use of artificial intelligence (AI) in medical imaging and radiotherapy has been met with both scepticism and excitement. However, clinical integration of AI is already well-underway. Many authors have recently reported on the AI knowledge and perceptions of radiologists/medical staff and students however there is a paucity of information regarding radiographers. Published literature agrees that AI is likely to have significant impact on radiology practice. As radiographers are at the forefront of radiology service delivery, an awareness of the current level of their perceived knowledge, skills, and confidence in AI is essential to identify any educational needs necessary for successful adoption into practice.

**Aim:** The aim of this survey was to determine the perceived knowledge, skills, and confidence in AI amongst UK radiographers and highlight priorities for educational provisions to support a digital healthcare ecosystem.

**Methods:** A survey was created on Qualtrics® and promoted via social media (Twitter®/LinkedIn®). This survey was open to all UK radiographers, including students and retired radiographers. Participants were recruited by convenience, snowball sampling. Demographic information was gathered as well as data on the perceived, self-reported, knowledge, skills, and confidence in AI of respondents. Insight into what the participants understand by the term “AI” was gained by means of a free text response. Quantitative analysis was performed using SPSS® and qualitative thematic analysis was performed on NVivo®.

**Results:** Four hundred and eleven responses were collected (80% from diagnostic radiography and 20% from a radiotherapy background), broadly representative of the workforce distribution in the UK. Although many respondents stated that they understood the concept of AI in general (78.7% for diagnostic and 52.1% for therapeutic radiography respondents, respectively) there was a notable lack of sufficient knowledge of AI principles, understanding of AI terminology, skills, and confidence in the use of AI technology. Many participants, 57% of diagnostic and 49% radiotherapy respondents, do not feel adequately trained to implement AI in the clinical setting. Furthermore 52% and 64%, respectively, said they have not developed any skill in AI whilst 62% and 55%, respectively, stated that there is not enough AI training for radiographers. The majority of the respondents indicate that there is an urgent need for further education (77.4% of diagnostic and 73.9% of therapeutic radiographers feeling they have not had adequate training in AI), with many respondents stating that they had to educate themselves to gain some basic AI skills. Notable correlations between confidence in working with AI and gender, age, and highest qualification were reported.

**Conclusion:** Knowledge of AI terminology, principles, and applications by healthcare practitioners is necessary for adoption and integration of AI applications. The results of this survey highlight the perceived lack of knowledge, skills, and confidence for radiographers in applying AI solutions but also underline the need for formalised education on AI to prepare the current and prospective workforce for the upcoming clinical integration of AI in healthcare, to safely and efficiently navigate a digital future. Focus should be given on different needs of learners depending on age, gender, and highest qualification to ensure optimal integration.

## Introduction and Background

### The AI Accelerating Trajectory

In the last decade, Artificial Intelligence (AI) implementation has accelerated but has also become an increasingly divisive topic in medicine, particularly so within medical imaging. The development of more sophisticated computers with greater storage capabilities and faster graphics processing units (GPUs) have allowed systems architectures to develop in a way which was not possible before (1). This has allowed convolutional neural networks (CNNs) in image recognition tasks to develop. These systems learn iteratively until acceptable performance is achieved relative to the previous interpretive standard (2). Wider availability of large medical imaging datasets and advancements in neuroscience further perpetuated AI technology advancement (3).

While AI is considered to be a promising, fast changing area of healthcare innovation (4), able to revolutionise care delivery, it is often seen with suspicion and mistrust by many healthcare professionals working in radiology, leaving them concerned about their future careers (5–7). In response to the impending digital healthcare revolution, the NHS has prioritised the development, testing, and validation of AI tools and digital health systems as part of their long-term improvement plan (8).

### AI Implementation Creates Controversy Among Medics, Including Radiologists

Despite these technological advances, implementation of AI into the clinical setting has been perceived differently across the multidisciplinary team. Difference research projects surveyed radiologists and radiology trainees, the medical practitioners within medical imaging. In 2019, Waymel et al. (9) surveyed 270 senior radiologists and radiology registrars in France and reported an optimistic view as clinicians felt that implementation of AI will have a positive impact on clinical practise. Respondents thought that AI will speed up reporting turnaround times, i.e., the time taken to produce a clinical diagnostic report, with a possible reduction in the number of imaging-related medical errors and subsequent increased contact time to enable more direct patient care. Further work by Oh et al. in Korea (10), surveyed the confidence of 669 doctors and medical students when using AI, where 62% of respondents reiterated the perception that AI would speed the collection of clinical data. In Germany, 83% of 263 surveyed medical students felt that AI will never replace the radiologist (11) however this is contradicted by reports ranging from 26 to 78% of respondents (doctors, nurses, and technicians) fearing that AI could replace them in their clinical role (10–13). A lack of trust and acceptance of AI systems is also apparent in the literature (14, 15) with results in Korea reporting that 79% of respondents would always favour the doctor's opinion over the AI when a conflict arose. Whilst in Germany (10), 56% of 263 surveyed medical students, stated that AI would not be able to establish a definitive diagnosis (11). The perceived advantages of AI in the current evidence-base are clear; however contradictory views exist internationally on how exactly AI will work in the clinical arena and whether it will lead to role depletion among physicians/healthcare workers and students.

### The AI Training Gap May Challenge AI Implementation Among Clinicians and Perpetuate Long-Standing Workforce Shortages

The majority of published literature has further highlighted a lack of training to empower healthcare practitioners to optimally use the capabilities of AI, as well as the lack of regulatory frameworks of AI-enabled healthcare products (16, 17) and lack of thorough scrutiny of reported studies, ensuring a robust knowledge base (18). The majority of physicians feel they have received insufficient previous information on AI and would consider attending continuous medical education or technically advanced training on AI, if available (9–12). Similarly medical students have reported either no AI training at all or insufficient training in AI with many believing it should be taught at undergraduate level and be part of the compulsory curriculum (11, 19).

Lack of adequate training on AI to prepare clinicians and explain basic AI concepts and applications may impact on the number of physicians choosing to specialise in radiology after graduation, as was highlighted by recent research in the UK (20). A total of 19 medical schools participated in a survey assessing attitudes of medical students toward AI, 49% of respondents reported that they would be less likely to consider specialising in radiology due to the impact of AI. A similar picture is emerging in the United States, where 44% of 156 survey respondents reported they would also be less likely to choose radiology as a specialty due to the influence of AI (13).

The lack of knowledge of AI benefits and risks and the skills gap on using AI tools by clinicians needs to be urgently addressed to cater for the workforce shortages in medical imaging and radiotherapy; the current Royal College of Radiologist statistics which state that “the NHS radiologist workforce is now short-staffed by 33% and needs at least another 1,939 consultants to meet safe staffing levels and pre-coronavirus levels of demand for scans” (20). This staffing shortage in medical imaging is further compounded by the College of Radiographers census of the diagnostic radiography workforce in the UK. Results reported that the average current UK vacancy rate across respondents was 10.5% at the census date of 1 November 2020 (21). It is imperative to use dedicated educational provisions to dispel the misperception that “AI will replace radiology staff, or that AI may deter staff from specialising in the role in the first place.” Further training is required not only on how to use AI itself but also on the advantages, challenges, and issues surrounding AI implementation into clinical departments to ensure the confidence of clinicians interested into these careers increases.

### The Impact of AI on Radiographers

Radiographers are registered healthcare professionals who work predominantly and directly with patients, families, carers, and service users but very closely with Radiologists. In the UK, diagnostic and therapeutic radiographers form the largest proportion of the workforce in clinical imaging (radiology) and radiotherapy departments, respectively. There are more than 30,000 members of the radiographers' professional body, the Society of Radiographers (SoR) (2020) (22), and 36,941 currently registered with the regulator for health and care professions, the Health and Care Professions Council in the UK (23). Collectively their roles encompass the provision of health screening services, clinical imaging for diagnosis, and imaging and therapeutic services to facilitate curative, palliative, surveillance, end of life, and forensic examinations. Radiographers interact with and care for thousands of people each day. This requires a wide and encompassing range of skills and knowledge and the ability to empower people in shared decision making. Radiographers work on the interface between technology and service users in clinical imaging and radiotherapy. They operate the equipment, produce, and report on diagnostic images.

Radiology and radiography, two interconnected but distinct professions, are traditionally considered to be early adopters of AI technology (24, 25), with computerised diagnosis used as early as the 1960s (8). Since then, there have been several periods of high activity in AI research and development with intervening periods of lower activity, so-called AI “winters” (26, 27). Pattern recognition computer aided diagnosis (CAD) tools have been part of mammography image interpretation since the 1980s (28, 29), some of which are extant today and perpetuate significant human input due to high false positive rates (14, 30).

While research related to radiologists' roles, clinical practise, and education in relation to AI has flourished, as discussed in the abovementioned paragraphs, very little research has considered the impact of AI on radiographers and their perception of using it in clinical practise. The limited literature available would suggest that radiographers are keen to engage with AI but controversy still exists whereby some radiographers feel that AI may deplete or threaten their jobs in the future whilst others think it may lead to more advanced role developments (31–34). Abuzaid et al. (35) surveyed the opinions of 34 radiologists and 119 radiographers in the UAE on their willingness to accept AI into practise. Staff were excited and ready to embrace AI, however 17% of respondents stated they had no knowledge of AI, 40% were self-taught, and 73% reported difficulty accessing training courses to fill the knowledge gap for staff. Further work by Botwe et al. (36) surveyed 151 radiographers in Ghana. Most respondents (83%) were positive and would embrace the implementation of AI into practise, however 83% expressed concerns about AI related errors and job displacement. A further 69% felt that AI could lead to reductions in radiation dose whilst maintaining image quality. Overall, they concluded that there was a need for further education for radiographers to alleviate these fears. Similar fears and apprehensions regarding trust and knowledge gaps have been expressed by radiographers in Canada, America, and Ireland (32–34). In particular the survey of 318 diagnostic and 77 therapeutic radiographers from Ireland has identified resistance of AI use in particular for patient facing roles. Respondents felt that radiographers would always have a primary role when caring for the patient and that AI would not be able to replace that human touch. Similar to other studies, >50% respondents worried about changing roles and fewer jobs for radiographers, as AI will take over clinical delivery. However this notion of role depletion was not universally supported in this survey as 47% of diagnostic and 38% of therapeutic radiographers felt AI will create new specialised/advanced roles in the future. This may mean the radiographers can work together with AI tools to fulfil roles that address the ongoing staff shortages.

### The Future of AI Within Medical Imaging and Radiotherapy: Challenges and Opportunities for Integration and the Importance of Education

Sarwar et al. (37) have predicted the full integration of AI in healthcare in the next 5–10 years. Implementation of AI into the clinical setting is not without barriers; these include a lack of trust and acceptance of the systems offered (9, 29), lack of training to empower healthcare practitioners to optimally use the capabilities of AI, as discussed above, the lack of standardised regulatory frameworks of AI enabled healthcare products (10, 12) and lack of thorough scrutiny of reported studies, ensuring a robust knowledge base (15) to name just a few. It is essential for the design, validation, and adoption of AI that radiographers are knowledgeable, competent, confident, and well-trained to be able to fully materialise the benefits of new technology while minimising risks but also to be in position to explain these benefits and risks to the patients; thus radiographers could be contributing to and sustaining of a safe, efficient medical imaging and radiotherapy service, one that is based on trust and research evidence on the use of appropriate AI technology.

A number of suggestions to allow AI systems to make their way into clinical application have been offered, such as various measures to make AI more interpretable or explainable (38, 39). The users of AI technologies, for instance the radiographers, radiologists, and oncologists and those responsible for the procurement of AI for healthcare, need to have adequate knowledge, and understanding of the functionality and applications of the proposed systems to enable unbiased selection, i.e., the best application choice for the intended function with an awareness of potential limitations and risks.

The Topol review (40) reiterates the need for education in AI to be integrated into preregistration programmes, and for the necessity of upskilling the existing workforce in AI applications and technology. Recent draught HCPC guidelines (41) state that radiographers should “be aware of the principles of AI and deep learning technology, and the methods of assessing the performance of AI algorithms” (p. 45). Recent recommendations and standards jointly delivered by the International Society of Radiographers and Radiological Technologists (ISRRT) and European Federation of Radiographer Societies (EFRS) (42), state that radiographers need to have functional and performance assessment knowledge of AI systems. This can be described as a form of “AI literacy” that should be included in both pre and post registration programmes, along with education for the whole workforce. The Society and College of Radiographers' AI Working Party has also recently offered recommendations for education and training of radiographers on AI theory and applications (43).

### Rationale, Aims, and Objectives

The paucity of information available on radiographers perceptions of AI and its implementation into daily clinical practise provides a strong rationale for the design of a dedicated study. As identified by Lai et al. (44) AI in healthcare will only be accepted and satisfactory for everyone, if we invest on collaborative effort and include everyone within the multidisciplinary team in the decision-making process. Hence, this exploratory study aims to highlight the perceived, self-reported, knowledge, skills, and confidence of UK diagnostic and therapeutic radiographers in relation to AI. Further objectives were to investigate the adequacy of training provisions currently available and to propose content and format of further education on AI.

## Methods

### Questionnaire Design

A questionnaire was designed using the Qualtrics® survey platform. This is an online survey builder which allows for open dissemination via an internet link, hence optimising participant reach (45). This voluntary, fully online survey was designed and reported to adhere to the Checklist for Reporting Results of Internet E-Surveys (CHERRIES) (46) and approved by City, University of London, School of Health Sciences Research Ethics Committee (ETH1920-1989). No incentives were offered to complete this survey.

This was a fully open survey, available from the 12th of February 2021 to the 6th of April 2021, for everyone who had the link. The survey was set to collect fully anonymous responses, therefore neither IP addresses nor any other identifying information was collected from participants. An opening slide gave participants information on the study rationale and aim, provided information on current literature on the subject, informed participants of the approximate time commitment to complete the survey and gained consent to proceed. A final survey slide notified respondents of submission of responses, although a full review of responses was not given. Participants were permitted to freely navigate back to previous questions and allowed to save responses and finish the survey at a later time in order to maximise response completeness. All responses were included in data analysis, even if the survey was not complete. Time for completion of the survey was, therefore, not analysed.

The questions included in the survey were loosely based on a previous, unpublished, survey undertaken by one of the co-authors. These initial survey was further modified and expanded with new questions based on input from all listed authors, many of whom are members of the “Society and College of Radiographers Artificial Intelligence Working Party,” who have a range of senior clinical and academic experience. The survey content is drawing upon current research evidence as outlined in introduction, as well as from the themes presented on the Society of Radiographers (SCoR) AI guidance document for radiography professionals (43).

### The Survey Instrument

The questionnaire consisted of 91 questions in total, divided into five main sections or “blocks”—(i) participant demographics, (ii) AI knowledge, (iii) skills and confidence in AI (including questions on education provision), (iv) perceptions of the impact of AI on clinical practise (v) expectations for the future of radiography with AI and finally, (vi) the effect AI may have on image perception and reporting. Most questions were either multiple choice format, with some free text options to allow for more detailed responses or Likert scale questions. Only one question required a fully open response.

The demographic section included seven questions to gather data on the age, number of years' experience, highest academic qualification, region of the UK, clinical setting, and nature of current role. This information was requested to allow investigation of any relationship between these independent variables and dependent variables of knowledge, skills and confidence in AI. An eligibility filtering question placed at the beginning of the survey enquired if the respondent was a practising or student radiographer; this was to ensure that anyone other than a radiographer did not complete the survey. If the participant responded that they are not a radiographer, they were redirected to the end of the survey and no further data was collected.

Only the results of the first three sections of the survey (i–iii) are discussed in this paper; the remaining will be presented in different publications given space limit and richness of findings.

### Validity and Reliability of the Survey Instrument

For each new survey face and content validity are vital measures of quality (47).

#### Face and Content Validity

Face validity, a subjective measure which concerns whether or not the instrument appears to potential test takers to be assessing what it intended to measure (48) has been assessed and ensured for our study (in terms of feasibility, readability, consistency of formatting, the clarity of the language used), through the piloting phase of the survey (49). Content validity, “the degree to which items in an instrument reflect the content universe to which the instrument will be generalised” (50) was ensured by the design and review of this work by the SCoR AI working party and other AI radiography experts, the piloting with another team of AI experts with varying demographics and professional backgrounds and by being grounded on relevant research evidence, including the SCoR AI guidance document for radiographers, which outlined priorities for AI adoption within the radiography workforce in all areas of practise, including education (43). The validation of the questionnaire was conducted by a panel of experts in the medical imaging and AI field, which included 12 qualified, practising radiographers, academics, students, and clinical staff, with a range of clinical experience from <1 year to >20 years. This tested both the technical aspects of the survey format (face validity) as well as the suitability of the questions (content validity). Minor formatting issues involving difficulty in navigating to the next question were reported and fixed before final dissemination of the survey.

#### Internal Consistency

Cronbach's alpha was calculated *post-hoc* for the Likert scale questions of this instrument to be able to confirm internal consistency (47). Acceptable internal reliability was found for the scale questions for both professions (α = 0.736 and α = 0.777 for diagnostic and therapeutic radiography, respectively).

### Participants

This survey was intended to give a national (UK) perspective on perception of knowledge, skills, confidence, and educational needs of both the diagnostic radiography and therapeutic radiography workforce in the field of AI. All radiographers (student and trainees, practising and retired, academic, and researchers) across all sub-specialisation areas, including sonographers, were invited to participate. The survey was disseminated via LinkedIn® and Twitter® employing non-probability snowball, sampling (51), and widely shared by the authors through their radiography-specific professional networks, many of whom are members of the SCoR AI Working Party or hold different AI leadership positions within decision making agencies. Academic colleagues were also approached to distribute within radiography academic communities and students.

The link to the survey was also sent to the leads of many clinical centres throughout the UK for dissemination to all colleagues, therefore ensuring maximum reach to relevant parties.

### Data Analysis

The IBM SPSS (version 23) was used for analysis of the data (52). Descriptive statistics, in the form of frequencies have been reported for most of the responses. One question required an open-ended response, which has been analysed by thematic content analysis, using NVivo (version 12) (53). Descriptive and inferential statistics were calculated using SPSS and graphs produced within MS Excel® (Microsoft, 2018). Data was presented in percentages for single response questions and counts/frequency for questions where more than one response was permitted. There were no weightings applied to any questions for analysis.

Combinations of some of the variables have been analysed to determine if any patterns emerged in order for hypotheses to be proposed for future studies (54). The correlations of independent variables such as: years practising, highest academic qualification, and age with dependent variables such as: understanding of AI, confidence in AI, understanding of the underlying terminology of AI, feelings of being well-trained in AI, and agreement that they have developed some skill in AI, were all explored and measured on either four-point or seven-point Likert scales, with the exception of “understanding of AI,” which was measured on a scale of zero to ten. Spearman's rank (*r*s) and Kendall's tau-b (*v*) correlations between ordinal data were run using IBM SPSS® (55). Responses which did not fit with the ordinal classification of the data were recategorised as “missing” before calculation, such as level of highest qualification option “other” and years' experience options “I do not work in the clinical setting” and “I am in retirement.” Missing data were excluded pairwise, meaning that data could be included even if the respondent did not enter a response to some other question. Bootstrapping was activated for 1,000 samples at 95% confidence levels. Subgroup analysis was then carried out to better understand the reason for any statistically significant correlations between ordinal data.

Chi-square test for independence was run for comparisons between nominal and ordinal data. In many cases, assumptions necessary to allow accurate interpretation of the Pearson's chi-square were found to be violated, so the “likelihood ratio Chi-square” statistic was used as an alternative. The likelihood ratio compares the likelihood of obtaining the observed data compared to the likelihood of obtaining the data if there is no significant difference in the variables, i.e., the data which would have been observed if there is no statistically significant relationship between variables (*p* ≤ 0.05) (56). Cramer's *V* (*V*) was then performed to quantify the magnitude of any relationship.

The resultant cross tabulations were interrogated to identify any major differences between observed and expected counts within subgroups for significant findings. Subgroup analysis was then carried out to better understand the reason for any statistically significant correlations.

Thematic analysis using NVivo® was performed to analyse qualitative responses (52). Responses to the open-ended question “Can you describe the term Artificial Intelligence in your own words?” were read and coded. Codes were reread and collated into four key themes.

## Results

### Demographic Information

Cleaning of the data removed any blank responses from the initial participants. A total of 415 radiographers responded to the survey. Four participants selected the option of “no consent,” leaving 411 survey responses for analysis.

Of the total respondents, 66.4% stated that they were practising diagnostic radiography (*n* = 273), 14.4% were diagnostic radiography students (*n* = 59), 16.1% stated they were practising therapeutic radiography (*n* = 66), and 2.7% were therapeutic radiography students (*n* = 11). This calculated to an approximate 1:4 ratio of therapeutic: diagnostic radiographers, which broadly represents the UK workforce ratio of 3,794 therapeutic to 20,231 diagnostic radiographers (57). The most recent data from the HCPC, stated above, is not broken down into diagnostic and therapeutic radiography (23). Two respondents indicated they were practising both diagnostic and therapeutic radiography.

There were responses from throughout the regions of the UK with the exception of therapeutic radiographers in the Channel Islands (Table 1).

**Table 1:** Respondents' demographic details presented as percentages (%) and frequencies (n).

		**Diagnostic radiography**	**Therapeutic radiography**
**Region of UK where respondents currently work**	England	56.7 (*n* = 183)	88.2 (*n* = 67)
	Scotland	30 (*n* = 97)	9.2 (*n* = 7)
	Wales	1.9 (*n* = 6)	1.3 (*n* = 1)
	Northern Ireland	11.1 (*n* = 36)	1.3 (*n* = 1)
	Channel Islands	0.3 (*n* = 1)	0 (*n* = 0)
**Years practising radiography**	0–2 years	22.7 (*n* = 75)	23.4 (*n* = 18)
	3–5 years	10.6 (*n* = 35)	16.9 (*n* = 13)
	6–10 years	13.9 (*n* = 46)	11.7 (*n* = 9)
	11–20 years	23.0 (*n* = 76)	23.4 (*n* = 18)
	>20 years	27.5 (*n* = 91)	22.1 (*n* = 17)
	Not practising	1.2 (*n* = 4)	1.3 (*n* = 1)
	Retired	1.3 (*n* = 4)	1.3 (*n* = 1)
**Age range**	18–25 years old	19.3 (*n* = 63)	23.7 (*n* = 18)
	26–35 years old	28.4 (*n* = 93)	26.3 (*n* = 20)
	36–45 years old	27.2 (*n* = 89)	25.0 (*n* = 19)
	46–55 years old	12.5 (*n* = 41)	18.4 (*n* = 14)
	56–65 years old	11.3 (*n* = 37)	6.6 (*n* = 5)
	>65 years old	1.2 (*n* = 4)	0 (*n* = 0)
**Highest academic qualification**	A-level	14.9 (*n* = 48)	11.8 (*n* = 9)
	BSc	24.2 (*n* = 78)	35.5 (*n* = 27)
	PgCert	19.9 (*n* = 64)	1.3 (*n* = 1)
	PgDip	13.0 (*n* = 42)	6.6 (*n* = 5)
	MSc	19.6 (*n* = 63)	36.8 (*n* = 28)
	PhD/EdD/DProf or equivalent	1.9 (*n* = 6)	3.9 (*n* = 3)
	Other	6.5 (*n* = 21)	3.9 (*n* = 3)
**Clinical setting/counts (respondents were permitted more than one selection)**	University teaching hospital	*n* = 195	*n* = 50
	District general hospital	*n* = 103	*n* = 19
	Private sector	*n* = 12	*n* = 2
	Poly-trauma unit	*n* = 30	*n* = 0
	Mobile unit	*n* = 4	*n* = 0
	Other	*n* = 14	*n* = 5
	I do not work in the clinical setting	*n* = 25	*n* = 4
**Current role**	Assistant practitioner radiographer	1.2 (*n* = 4)	0 (*n* = 0)
	Undergraduate radiography student	19.6 (*n* = 63)	13.2 (*n* = 10)
	Clinical radiographer	39.1 (*n* = 126)	38.2 (*n* = 29)
	Research radiographer	0.9 (*n* = 3)	2.6 (*n* = 2)
	Advanced practitioner	15.8 (*n* = 51)	17.1 (*n* = 13)
	Ph.D. researcher radiographer	0.6 (*n* = 2)	0 (*n* = 0)
	Other	3.1 (*n* = 10)	6.6 (*n* = 5)
	Academic in radiography: teaching only	0.9 (*n* = 3)	1.3 (*n* = 1)
	Industry partner	0.3 (*n* = 1)	1 (*n* = 0)
	Consultant radiographer	4.3 (*n* = 14)	13.2 (*n* = 10)
	Clinical academic/Lecturer: practitioner	3.1 (*n* = 10)	1.3 (*n* = 1)
	Radiology/Radiographer/Radiotherapy manager	6.2 (*n* = 20)	6.6 (*n* = 5)
	Retired radiographer	0.9 (*n* = 3)	0 (*n* = 0)
	Academic in radiography: teaching and research	3.7 (*n* = 12)	0 (*n* = 0)
***Diagnostic radiography*** **sub-specialism/counts (respondents were permitted more than one selection)**	General radiography including emergency, theatre, and fluoroscopy	*n* = 207	
	Mammography	*n* = 32	
	MRI	*n* = 56	
	CT	*n* = 100	
	Ultrasound	*n* = 25	
	Interventional	*n* = 44	
	PET/CT	*n* = 3	
	PET/MRI	*n* = 1	
	DEXA/DXA	*n* = 5	
	Reporting	*n* = 63	
	Radiology manager	*n* = 20	
	PACS administrator	*n* = 9	
	Education	*n* = 54	
	Policy maker/professional advocate	*n* = 11	
	Other (diagnostic)	*n* = 22	
***Therapeutic radiography*** **sub-specialism/counts (respondents were permitted more than one selection)**	Pre-treatment, simulation, contouring, immobilisation		*n* = 35
	Treatment planning		*n* = 15
	Treatment delivery		*n* = 54
	Patient information/support/review		*n* = 23
	Educator		*n* = 7
	Research		*n* = 7
	Management		*n* = 10
	Quality assurance/Quality improvement		*n* = 7
	DEXA/DXA clinical applications		*n* = 0
	Other (therapeutic)		*n* = 7

A range of years of experience was indicated in both diagnostic radiography and radiotherapy. Visual inspection would indicate there is a similar distribution amongst respondents in each profession (Table 1).

There was representation across all age groups except for the over 65 years old group in radiotherapy (Table 1).

Of the diagnostic radiography respondents (including students), 26% indicated they were male, 72.2% female, 0.6% non-binary/third gender, and 1.2% preferred not to say. This is similar to the radiotherapy respondents of whom 22.4% responded that they were male and 77.6% female, which is broadly representative of the UK radiographer workforce, which has an approximate 1:3 ratio of male to female (47).

#### Highest Academic Qualification

For both diagnostic radiography and therapeutic radiography, most respondents indicated their highest level of academic qualification as a BSc, with 24.2 and 35.5%, respectively. There were fewer diagnostic radiographers who have attained a MSc (19.6 and 36.8%) or doctoral level qualification (e.g., Ph.D., Ed.D.) (1.9 and 3.9%) than therapeutic radiographers, respectively. Those with A-level or equivalent are assumed to be student radiographers. This data is represented in full in Table 1. Those who selected “other” were asked for further explanation, with the majority of the respondents across both professions stating they hold a Diploma of the College of Radiographers (DCR) (*n* = 7). Other responses included conversion degrees such as MRad (*n* = 2), or other types of master's degrees such as MEd (*n* = 1) and MA (*n* = 2).

#### Clinical Setting

The greatest proportion of participants from both professions indicated that they work in university teaching hospitals, closely followed by the district general hospital setting. Full details of other responses are given in Table 1.

For those who responded “other” in therapeutic radiography, two stated they worked in a foundation trust, three in a specialist cancer centre, two were students, and one stated they were a university lecturer. Most free text responses from the diagnostic radiography participants indicated that they worked in the university setting as either an academic or researcher (*n* = 15), followed by responses from students (*n* = 10).

#### Role Description

Most of those in clinical practise from both professions indicated that they were practising as a clinical radiographer (39.1 and 38.2%, diagnostic radiography and radiotherapy, respectively), followed by those choosing the “advanced practitioner” option (15.8% and 17.1%, diagnostic radiography and therapeutic radiography, respectively). There are fewer consultant radiographers responding to this survey in diagnostic than therapeutic radiography (4.3 and 13.2%, respectively), although it should be noted that there were more options available for the diagnostic radiography respondents. This was to best reflect the specific career landscape in both professions (Table 1).

#### Area of Expertise/Sub-Specialism

Respondents were given the option of selecting up to three options from the list, along with a free-text option for further explanation. Most diagnostic radiographers indicated that they were involved in general radiography (32%) followed by CT (15%), followed closely by those working in reporting, MRI and education. The responses from respondents in the radiotherapy cohort indicated that the majority were involved in treatment delivery (33%), followed by pre-treatment, simulation, contouring, and immobilisation (21%) (Table 1).

From those who selected “other” in diagnostic radiography, most responses were cardiac catheterisation (*n* = 4) and nuclear medicine (*n* = 3). Radiotherapy respondents indicated areas of sub-specialism in breast cancer (*n* = 1), research (*n* = 1), stereotactic radiosurgery (*n* = 1), and Information management and technology (*n* = 1).

### Perceived Knowledge, Skills, and Confidence in AI

An understanding of perceived knowledge, skills and confidence in AI was sought through an open question, asking respondents to describe the term “artificial intelligence” in their own words and a number of Likert-scale questions.

#### Understanding of the Term “Artificial Intelligence”

Responses were initially coded using thematic analysis for each of the professions, resulting in 21 codes (Supplementary Table 1; Supplementary Figure 1). Most codes were common across both professions (Supplementary Figure 2). Four general themes emerged from thematic analysis: (i) clinical applications of AI, (ii) advantages of AI, (iii) disadvantages of AI, (iv) technical information of AI technology (Supplementary Table 1).

The top three most frequent codes in the responses from the diagnostic radiographers' cohort included:

(i) understanding of AI as used in the identification of pathology or abnormality (clinical applications), for example the following quotes are presented as relevant;
  “reporting, without a practitioner looking at the film. Used to detect cancers…”
  “…report diagnostic images”
(ii) statements regarding the AI tasks which would normally require human input for example, the following quotes are presented as relevant;
  “…automated use of computers to perform human tasks.”
  “…computer algorithms performing tasks that usually rely on human interaction.”
(iii) comments with evidence of deeper understanding of “modern” AI systems, such as descriptions of systems which learn from example and “computer vision” for example the following quotes are presented as relevant;
  “…machine learning.”
  “…can be programmed to develop themselves on their own writing their own code, developer might even cease to understand the code.”

The top three codes from the therapeutic radiographers' responses were similar, with the majority of comments relating to:

(i) changing radiography workflows (AI replacing or augmenting tasks which require human input) for example the following quotes are presented as relevant;
  “…the use of technology, reporting, and verify systems, treatment planning systems to support patient pathway.”
(ii) technical description of “modern” AI systems, for example the following quotes are presented as relevant:
  “…use of computer algorithms to do mundane tasks e.g., outlining organs at risk (OAR).”
  “The use of complex interconnecting self-designing algorithms to achieve a specific outcome…”
(iii) clinical applications of AI in radiotherapy, such as segmentation, planning, and/or contouring. The following quotes are presented as relevant:
  “Automated RT planning to standardise planning”
  “Using software algorithms to calculate/determine outcomes previously determined manually, such as auto-contouring…”

Finally there were very few comments regarding the disadvantages of AI systems in both professions, with only two comments from diagnostic radiography and one from the therapeutic radiography cohort. A representative quote from the diagnostic radiography is noted below:

“Its current role is very ‘task dependent' and limited as it struggles to understand poor quality images, artefacts, or normal variants, or post-surgery image appearances, often it is classed the ‘next best thing' but most likely it is the new ‘emperors clothing”'
  Another representative comment was offered by the radiotherapy respondents:
  “Human reliance on technology… create(s) more work to me at work for simple decision-making process.”

#### Perceived Knowledge and Understanding of AI Terminology

**Figure 1:**
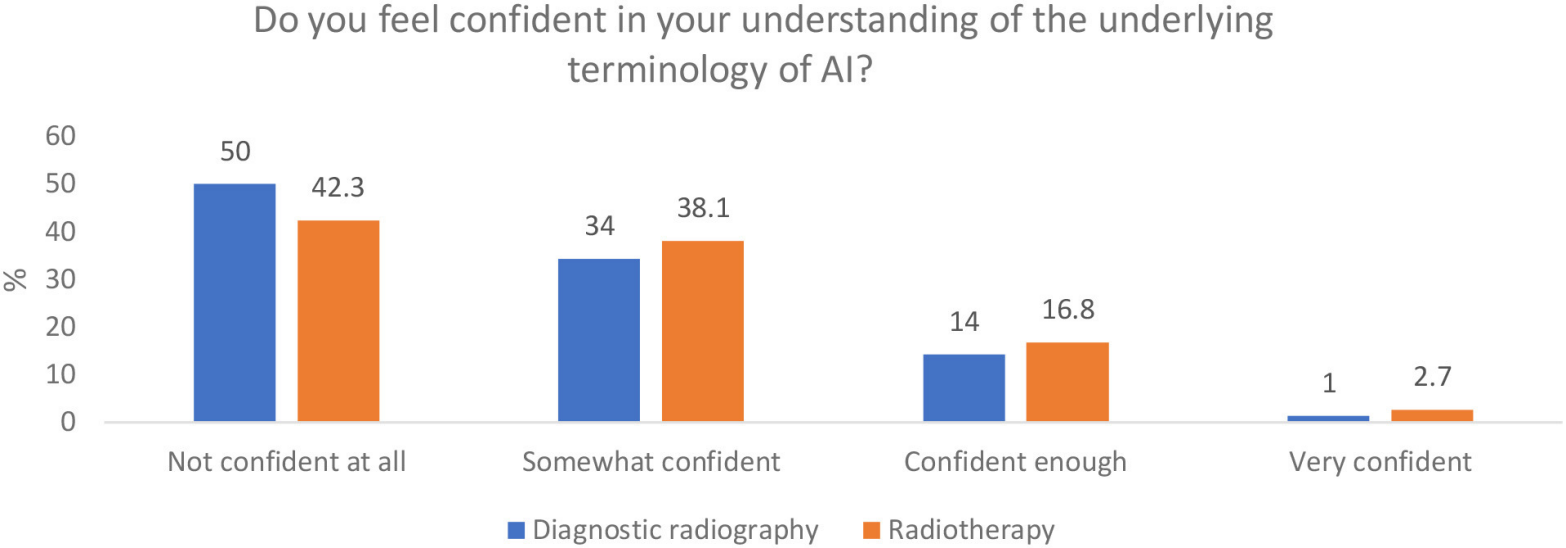
Respondents' understanding of the terminology of modern AI.

Examples of terms associated with modern AI technology and development were provided in the question represented in Figure 1—algorithms, deep learning, neural networks, computer-aided detection diagnosis, data mining, and over-fitting. The results demonstrate that 42.3% of diagnostic radiography and 50% of radiotherapy respondents were not confident at all in the terminology used in AI.

#### Development of Skill in AI

**Figure 2:**
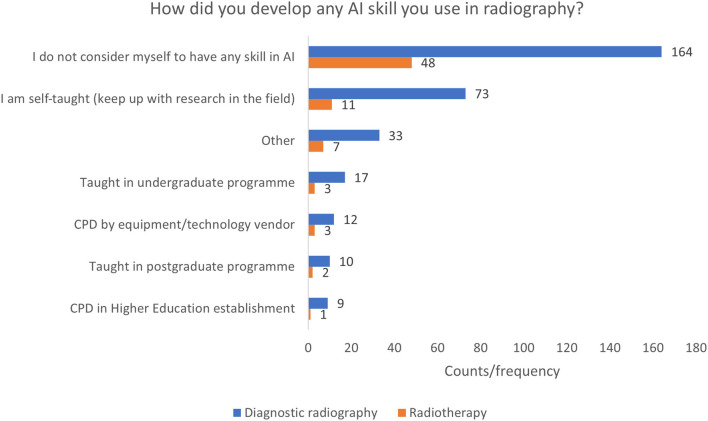
Development of skill in AI.

Most of both diagnostic radiography and radiotherapy respondents indicated that they do not feel they have developed any skill in AI used in radiography (51.6 and 64.0% of total responses, respectively) (Figure 2). Out of the other options presented, the majority in both professions indicated that any skill has been developed from their own, self-directed learning (21.0%). In both professions, the fewest responses came from the “CPD in a higher education establishment” option. The “other” option was selected by 40 respondents over the two professions. The diagnostic radiography respondents indicated that they have undertaken assignments or dissertations in AI (*n* = 8), have read around the subject or taken online courses (*n* = 4), have had equipment training or in house training (*n* = 4), contributed to a research project conducted by someone else (*n* = 3), listened to presentations at conferences (*n* = 3), or had some form of AI training integrated into a postgraduate qualification (*n* = 3). The radiotherapy comments included, workplace/applications training or through current use (*n* = 4), knowledge from a previous career (*n* = 1), and one respondent stated that they work for an AI company.

#### Confidence in Using AI in Radiography

**Figure 3:**
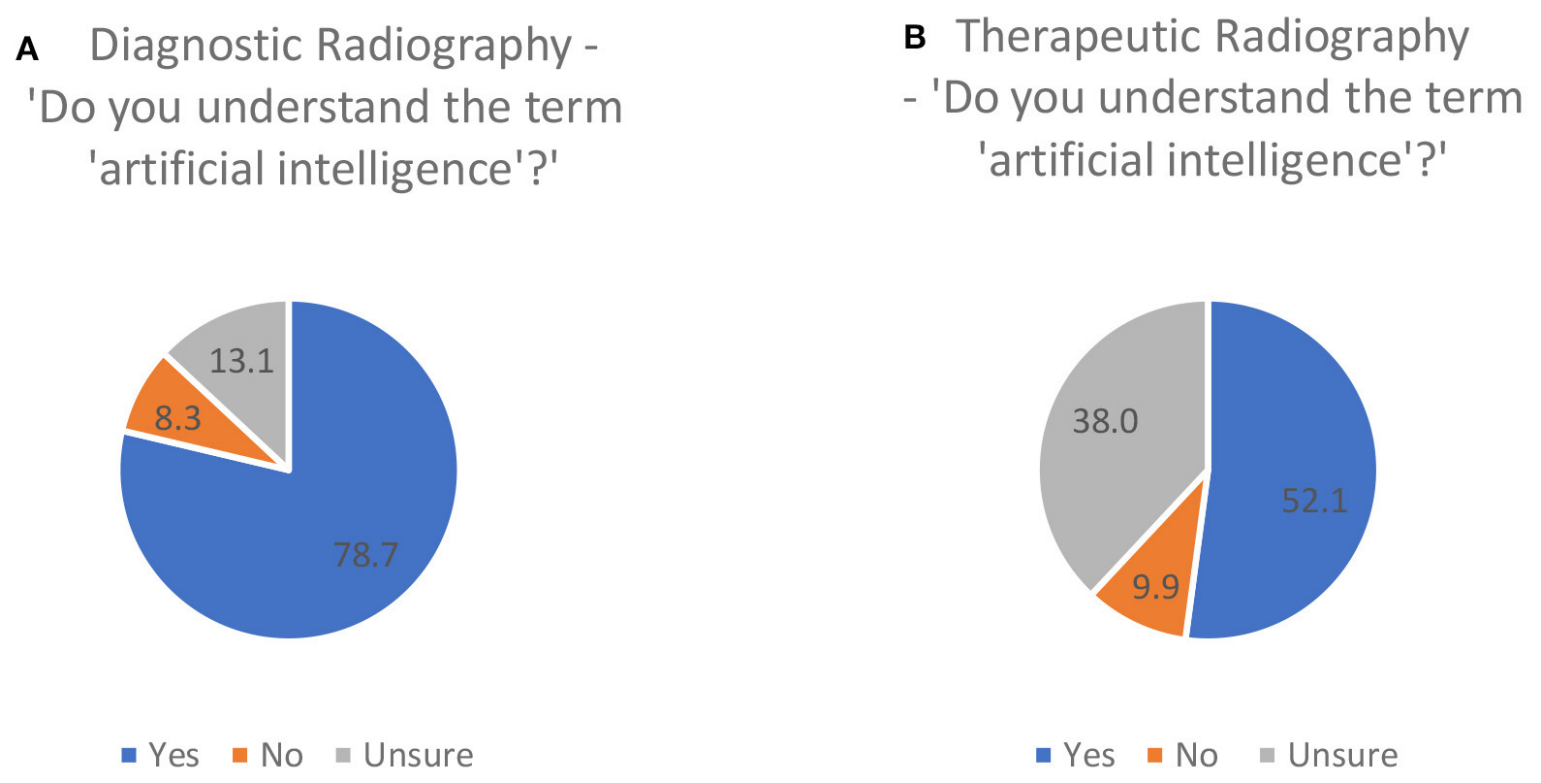
Respondents' perception of understanding of the term “artificial intelligence” [**(A)**, diagnostic radiography, **(B)** therapeutic radiography].

**Figure 4:**
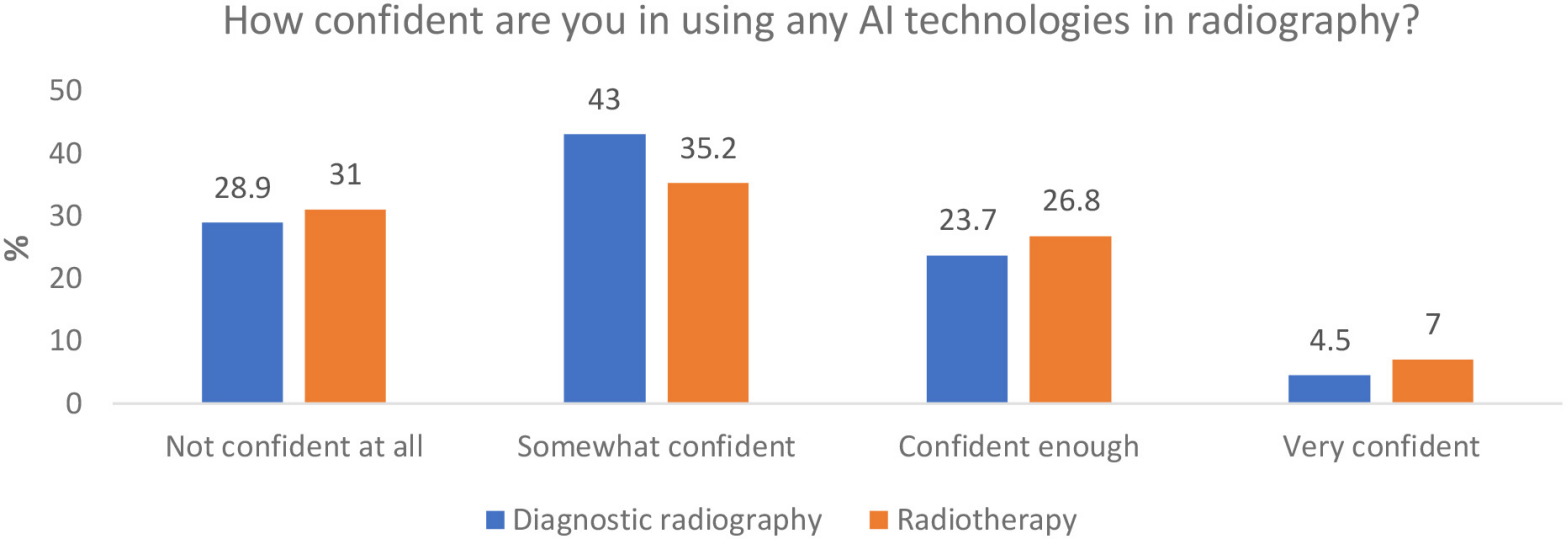
Respondents' perceived confidence in using AI technologies.

More of the diagnostic radiography respondents indicated that they understood the term AI than the radiotherapy respondents (yes, no, unsure) (78.7 and 52.1%, respectively) (Figures 3A,B), although a slightly smaller percentage of diagnostic radiographers stated that they felt confident in using AI technologies in radiography, compared to the radiotherapy responses (28.2 and 33.8% confident or very confident, respectively) (Figure 4). Respondents from both professions indicated a moderate understanding of AI and asked to rate it using a 0 to 10 scale, with 0 representing no knowledge at all and 10 representing “expert.” A mean response of 5.5 and 4.5 (0–10 scale) was reported for diagnostic radiography and radiotherapy, respectively.

### Perceived Acquired Skills in AI and Training to Support These Skills

Questions were posed to respondents regarding their perceived level of skill in AI, how they have developed this skill, the nature of any training they have received and how prepared they feel their skills or training has made them for the implementation of AI in the clinical setting.

#### Perception of Availability of AI Training for Radiographers (Generic)

**Figure 5:**
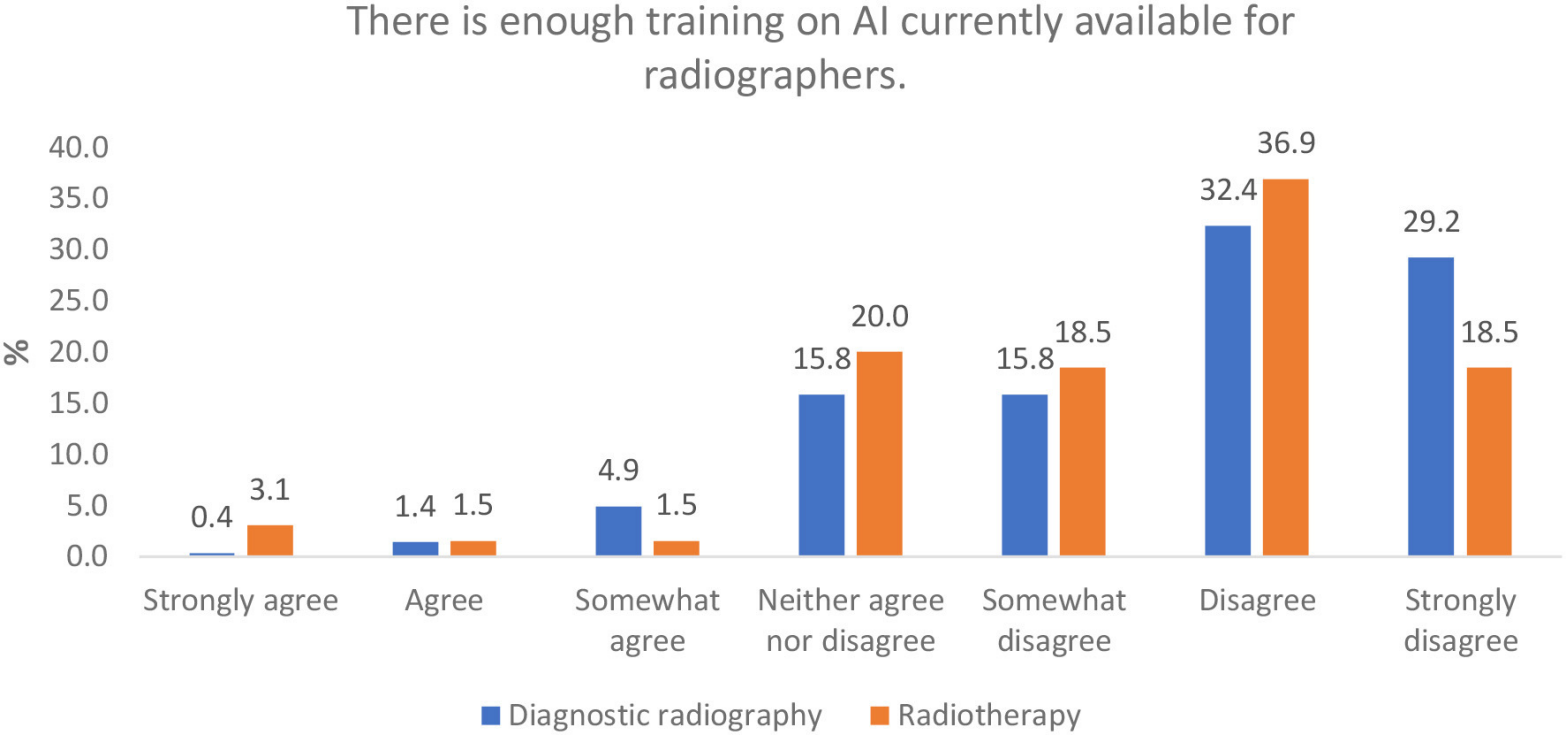
Perception of AI training availability for radiographers.

The majority of respondents from both professions either disagree or strongly disagree with this statement, with a “disagreement” aggregate (somewhat disagree, disagree, and strongly disagree) of 77.4 and 73.9% and an agreement aggregate (somewhat agree, agree, and strongly agree) of only 6.7 and 6.1% for diagnostic and therapeutic radiography, respectively (Figure 5).

#### Perception of Adequacy of Training Provisions for AI Implementation

**Figure 6:**
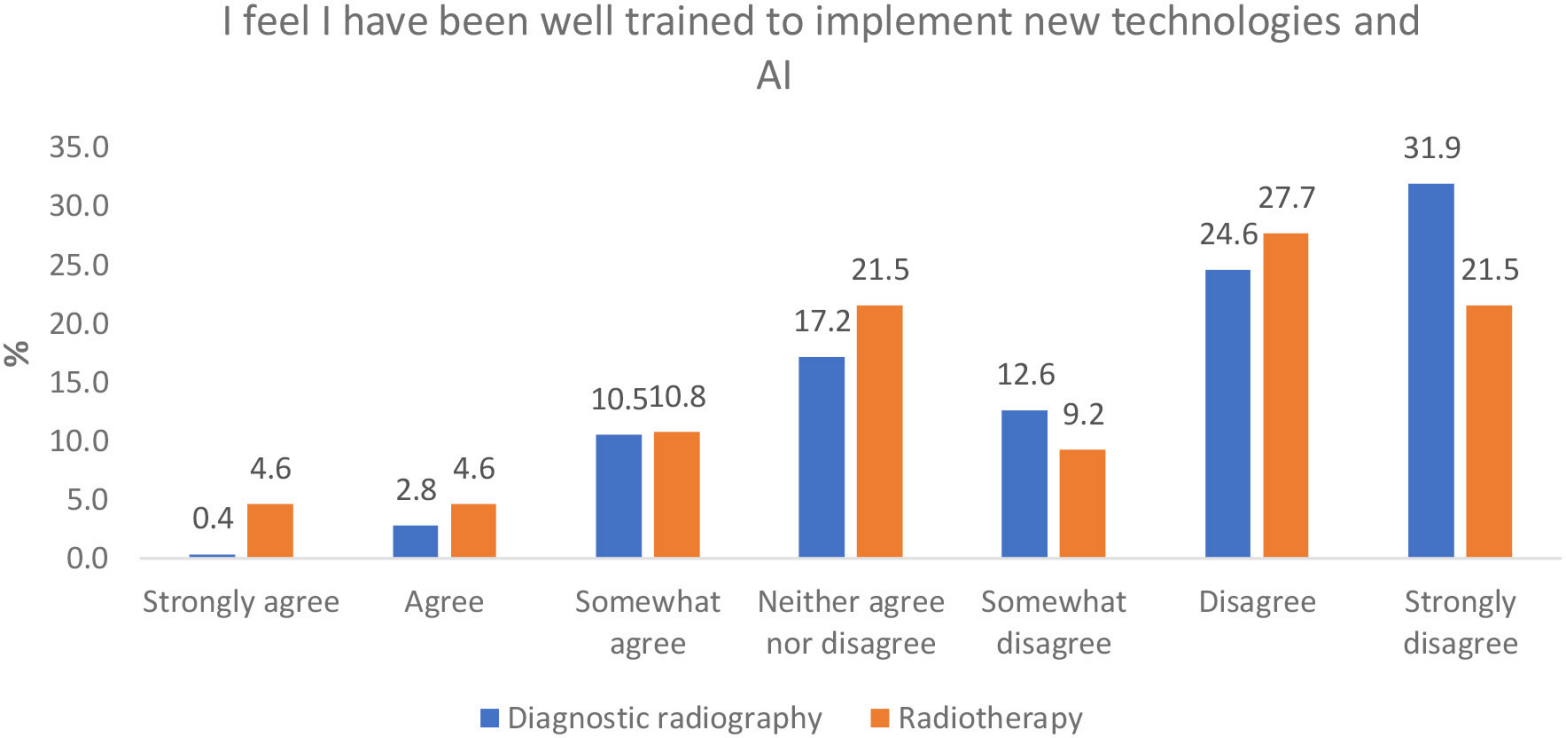
Perception of ‘adequacy of radiographers' AI training for clinical implementation.

Both professions indicated they did not feel well-trained to implement new technologies and AI, with over half (56.5%) of diagnostic radiography respondents indicating they either disagreed or strongly disagreed with this statement. This proportion was only slightly lower for radiotherapy (49.2%) (Figure 6).

#### Perception of Skill Acquisition in AI Clinical Applications

**Figure 7:**
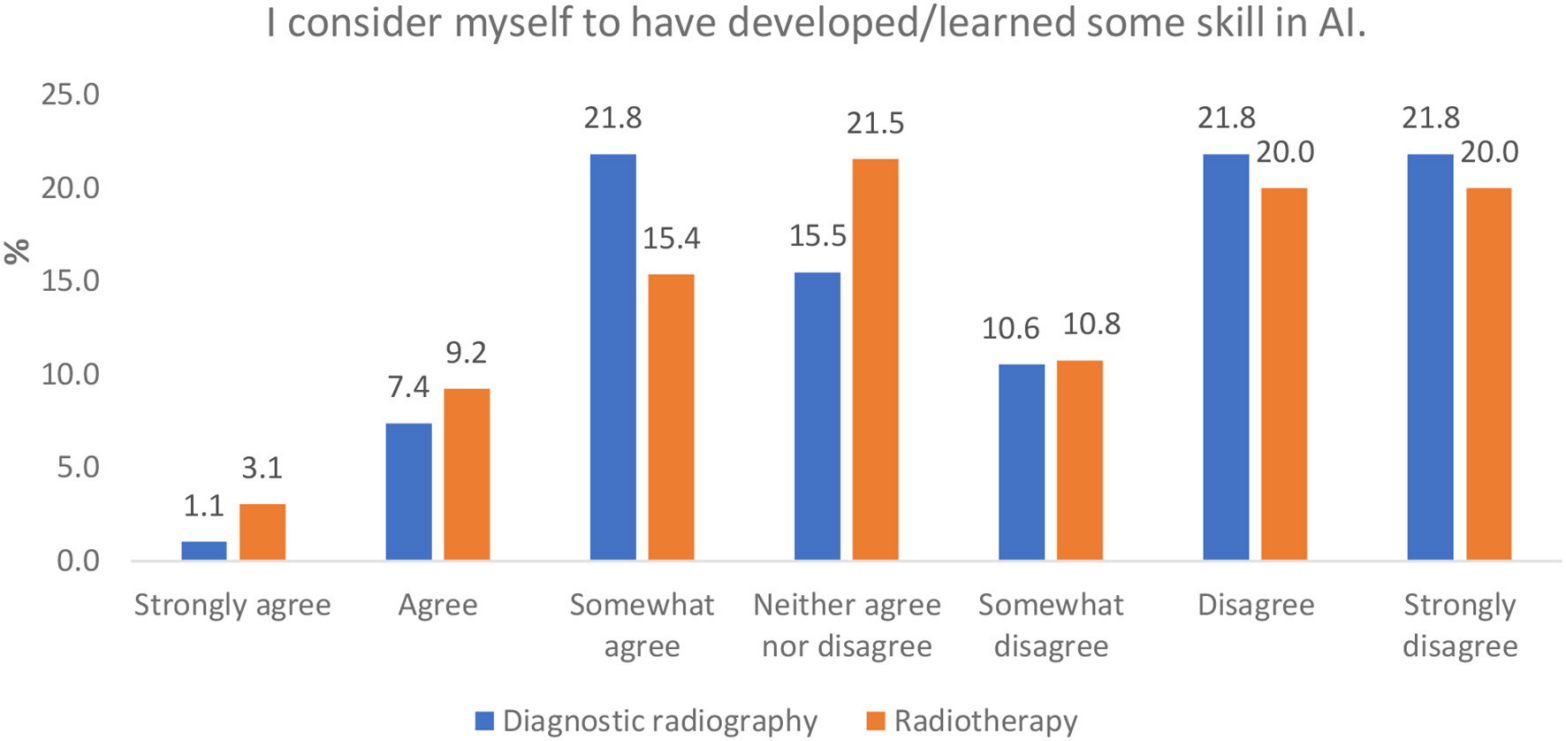
Perceptions of having developed/learned some skill in AI.

An aggregate of responses in the disagree categories (somewhat disagree, disagree, and strongly disagree) and agree categories (somewhat agree, agree, and strongly agree) from respondents in both professions indicate that they did not feel they had developed skill in AI, with “disagree” in diagnostic radiography being higher than “agree” (54.2 vs. 30.3%). This is similar to the radiotherapy responses (50.8 vs. 27.7%) (Figure 7).

### Future Training Content and Format on AI-Enabled Technologies

To determine the type of training and education requirements needed in radiography, two questions were asked. One question sought to gather information on the content of any training—what topic areas radiographers felt should be included in any training delivered, and another question on how or in what format this training might be best delivered in.

#### Topic Areas Needed for Training

**Figure 8:**
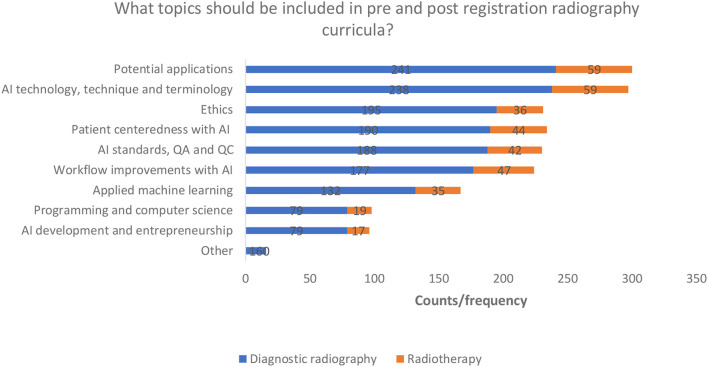
AI education topic preferences.

Most respondents from both professions indicated that they were interested in learning about potential applications of AI and AI technology, techniques, and terminology. Programming and computer science and AI development and entrepreneurship were not popular choices (Figure 8). The “other” option was chosen by 16 respondents from the diagnostic radiography cohort and mostly included comments suggesting uncertainty around what should be included. Two comments suggested that it is too early to consider any education in AI.

#### Training Format Preferences

**Figure 9:**
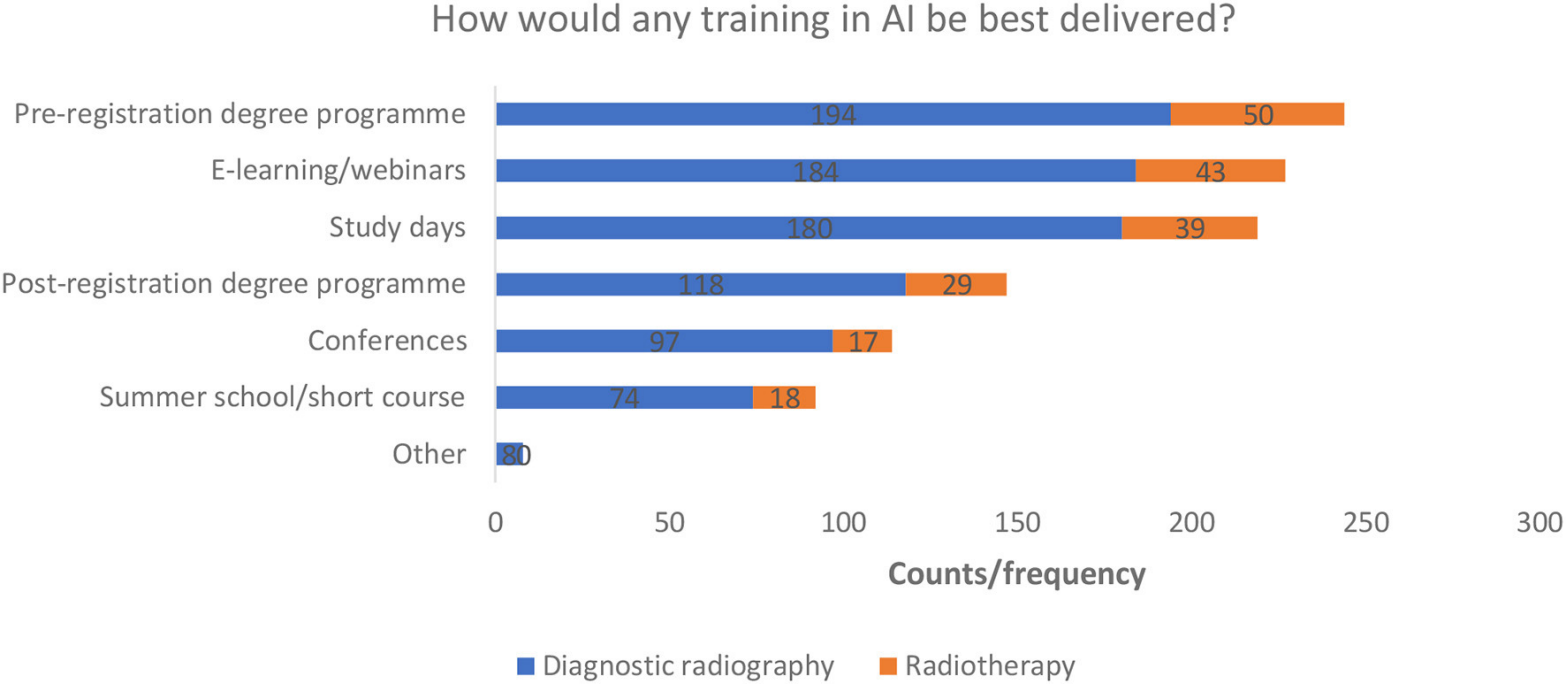
Training delivery preferences.

Most respondents indicated that training would be best delivered as part of a preregistration degree programme. E-learning/webinars and study days also received a high proportion of the total responses. All options were selected by some respondents (minimum respondent frequency *n* = 92 counts) (Figure 9). Eight diagnostic radiography respondents selected the “other” option. Suggestions included; annual CPD days for qualified staff and summer schools for pre and post registration radiographers to allow time for this training to take place in an already busy academic year.

## Comparisons

### Ordinal vs. Ordinal Comparisons

A selection of ranked variables (ordinal data) were compared using Spearman's rho (*r*s) and Kendall's tau (*v*) to identify any correlations. The results are presented in full in Supplementary Table 2. There was only one combination of variables which produced statistically significant results in both professions i.e., the relationship between highest level of academic qualification and understanding of AI on a scale of 0–10, where a medium strength positive correlation was found in both professions (54). Sub-group analysis revealed that for both the diagnostic and therapeutic responses, there was a general downward trend in the lower rating of confidence (i.e., scoring 0–3) as level of academic qualification increased, with the reverse apparent for the higher ratings of confidence (i.e., score of 7–10), i.e., as level of highest academic achievement increased, the number of respondents reporting higher levels of confidence increased. This data is presented in full in Supplementary Tables 3–6.

In the diagnostic radiography responses, there was also a significant positive relationship between highest level of academic qualification and confidence in AI terminology (*r*s = 0.151, *v* = 0.218, *n* = 271, *p* = 0.05), but this was not the case in the radiotherapy cohort. Further analysis of the groups reveals that very few respondents across all categories are very confident, or confident enough and a general downward trend in the “not confident at all” selection, i.e., as level of highest academic qualification increased, from undergraduate to Ph.D./Ed.D./D.Prof. or equivalent, the proportion of respondents indicating that they were “not confident at all,” decreased (Supplementary Table 7).

Additionally, a significant, medium strength positive association (*r*_s_ = 0.417, *v* = 0.313, *n* = 71, *p* = 0.01) was found in the radiotherapy responses between age and understanding of AI (scale 0–10) and respondents' years' experience and understanding of AI (scale 0–10) (*r*_s_ = 0.437, *v* = 0.332, *n* = 70, *p* = 0.01). This was not mirrored in the data obtained from the diagnostic radiography responses (Supplementary Table 2). Visual analysis of the subgroup data indicates that, there was a general downward trend in the lower rating of confidence (i.e., scoring 0–3) as both age category and years practising increased, with the exception of the 55–65 years age group, as demonstrated fully in Supplementary Tables 8–11.

There was no significant correlation in any of the other comparisons.

### Nominal vs. Ordinal Comparisons

There were no associations found between variables in the majority of tests, presented in full in Supplementary Table 12. There were four tests in diagnostic radiography and three tests in radiotherapy which showed a significant relationship between variables.

In both professions there was a statistically relationship between gender and the confidence in AI terminology, with a medium and large magnitudes in diagnostic radiography and therapeutic radiography, respectively.

Additionally, in diagnostic radiography, the “likelihood Chi-squared test” showed a significant relationship between:

(i) gender and confidence in using AI technologies a medium association strength, where male respondent report greater perceived confidence than females (Supplementary Table 12),
(ii) gender and confidence in the terminology of AI with a medium association strength, where male respondent report greater perceived confidence than females (Supplementary Table 12),
(iii) radiographers' role and their perceptions of the adequacy of training available, with a medium association strength, where perceptions of adequacy of training was lowest in the student radiographer responses (Supplementary Table 12), and
(iv) UK region and confidence in AI terminology with a small association strength, with no apparent pattern (Supplementary Table 12).

In radiotherapy, significant relationships were found to exist between:

(i) gender and understanding of AI with large association strength, where male respondent report greater perceptions of understanding than females (Supplementary Table 12),
(ii) gender and confidence in the terminology of AI, where male respondent report greater perceived confidence than females (likelihood ratio with a large association strength; Supplementary Table 12),
(iii) radiographers' role and understanding of AI with large association strength, where perceptions of understanding was lowest in the student radiographer responses (Supplementary Table 12).

## Discussion

The focus of this survey was to establish a “snapshot” of UK radiographers' perceived knowledge, skills and confidence in AI and to establish the specific detail of the educational need and preferences of this workforce, in line with AI radiography guidance and priorities (43). Furthermore, as an exploratory study it would help provide direction for future targeted AI research projects in the under-researched field of radiography.

### Perceived Knowledge, Understanding, and Confidence

Although a large proportion of both professions indicated that they understood AI in general, further specific responses from both professions made it clear that respondents were not very confident when using AI technologies. There was also a lack of understanding of the specific terminologies used in modern AI, such as “algorithms,” “deep learning,” “data mining,” “over-fitting,” and “neural networks” (Figure 1). This may indicate that, perhaps, initial reported “confidence” was surrounding AI in general rather than AI in radiography and modern AI. Abuzaid et al. (35) surveyed radiographers and radiologists in the United Arab Emirates (UAE) and found that 40% of respondents were not familiar with AI and a further 30% had merely a basic understanding. Other studies also report that there is a general lack of understanding of AI amongst radiologists (58, 59). The knowledge and understanding of AI at this level of detail is essential when engaging with literature around modern AI (60). Many applications of AI in medicine are currently in the development stage and therefore it is imperative for all clinicians to understand the literature in order to have a critical appreciation of the “potentials, pitfalls, and risks” of proposed technology as we move into the inevitable implementation phase (6).

### Level of Skill and Importance of Education and Training

A barrier to clinicians' confidence and understanding may be the dearth of education on the subject, with many radiographers in both diagnostic and therapeutic radiography stating that they do not consider themselves to have any skill in AI. Botwe et al. (36) conducted a survey of African radiographers on their perception of AI in diagnostic imaging and reported that 82.2% of 151 respondents felt that a lack of knowledge will be a significant barrier to the implementation of AI in the clinical setting. This is supported by the responses from our survey indicating that very few respondents felt that they were well-trained to implement AI and new technologies in the clinical setting and why both professions overwhelmingly agree that there is not enough education and training available in AI for radiographers (Figure 5). Abuzaid et al. (35) further support this in their survey of radiographers and radiologists in the UAEs, reporting that 74.5% of radiographers and radiologists responding to their survey had not studied AI as part of their degree, that 73.9% indicating that the availability of education and training will be a barrier to the implementation of AI and that 68.6% of clinical staff lack even a basic understanding of the technology.

As radiography is an evidence-based, applied science profession our day-to-day learning is supported formally, and informally, through our clinical placement and later on clinical roles (61). This is evidenced by the number of respondents, who reported that, despite not always having been formally trained, they did have some skill in AI, indicating that they had to seek out their own learning (Figure 2) and that AI has started to permeate radiography practise. Abuzaid et al. (35) concur, with 39.9% of respondents to their survey being self-taught in AI. Radiographers tend to learn to work with the tools which are introduced into the clinical setting, perhaps without the time or resources to fully understand the technology (62). This may have implications when newer, more complex forms of AI are introduced, which need to be approached more critically due to complex systems architectures and whose method of decision making are not so humanly interpretable (2, 15, 38). Being in position to know the theory behind the practise will enable healthcare professionals and radiographers to query, flag, escalate, and troubleshoot concerns in the functionality of AI ecosystems and intervene, as and when needed, with human intelligence, for the safety of the patients.

### Suggestions for the Type and Format of AI Learning

The radiographers responding to the survey indicate they wish to have education on potential AI applications, technology (technique and terminology), patient centeredness with AI, AI ethics, AI standards (quality assurance and control), and workflow improvements. These are areas which, perhaps, the workforce foresees or even witnesses as being the most impacted by AI (63). These may also be the areas that radiographers feel they can more easily relate to, and grasp given their training at level 6 (Bachelor's level) to allow for a smoother transition into a new field. Other proposed topics included applied machine learning, programming and computer science, and AI development/entrepreneurship, although these subject choices were less popular. The above list of topics is similar to those identified in the literature as important for inclusion in AI curricula, although it is also suggested that a more flexible curriculum should be offered to best suit the students' interest and current developments in the field (64, 65). A minority (2.5%) of respondents across both professions indicated that they had received training as part of a CPD programme in a higher education setting. This could lead to some national or global disparity and variability in the type and standard of education being delivered in AI knowledge in the future (35) and could impact speed and quality of AI adoption and implementation as well as job satisfaction. The development of a standardised or recommended AI curriculum, as suggested for radiology trainees, may provide a solution for this (16, 58, 59).

The respondents indicate that the best place for any AI training was in the pre-registration setting. This aligns with the proposed changes to the HCPC Standards of Proficiency (radiographers) which highlight the necessity for all radiographers to have an awareness of both the principles of AI, and of the methods of assessment of performance of any AI algorithm (41). If accepted, these changes would make it essential that all HCPC registrants and aspiring registrants have this knowledge, and therefore this learning must be front-loaded in the radiography education, in both the pre-registration as well as post-registration stages. The Topol review (40) supports this by recommending that training in digital technologies and computer science should be integrated into undergraduate education for health care professionals. A systematic review by Schuur et al. (16) examines training opportunities in AI for radiologists and found that there was an overwhelming prevalence of short courses offered, rather than those integrated fully into curricula, with education providers only involved in a limited capacity. Interestingly this is not fully supported in the results from our study which found that, although the respondents indicated they did not receive specific training in AI, there was a statistically significant relationship between the level of highest academic qualification and understanding of AI. This suggests that the higher the level of academic qualification, the greater the perception of understanding in AI. In the absence of specific AI training, this may be simply due to the way which postgraduate students are required to develop transferable skills as fully independent learners and the encouragement of those studying for higher academic qualifications to become agents of change and therefore actively investigate current and future developments (such as AI) for clinical practise themselves (66).

### Gender, Age, Qualification, and Role Correlations in Artificial Intelligence for Radiographers

The results from the analysis of the nominal data indicated that there is a relationship between gender and confidence in using AI terminology across both professions. Further exploration into the reason for this relationship were investigated from the cross tabulations of the likelihood ratios. This found that, on the whole, the observed values (responses) from the male respondents were higher than the expected values for “confident” and “very confident” and the female respondents were generally the reverse.

The reason for this is unclear, although it should be noted that there were fewer male respondents than female in both professions (approximately 1:3 male:female respondents from both professions, which is representative of the workforce gender distribution). Studies indicate that AI and computer science are male dominated fields (67), with only 18% of authors at AI conferences are considered female and that in general, females are less confident in using technology than males (68). This may be an issue for the radiography workforce, where there is a much greater proportion of females than males (57). This is in contrast to the radiology workforce demographics, where 60% of the workforce are male (69). According to the Dunner-Kruger effect (64), self-reported confidence is no measure of competence. A possible explanation for the lower confidence scores for women in our study may be due to the gender confidence gap and the tendency for women to think less favourably about their scientific reasoning ability and underestimate their performance (65).

Studies suggest that while there remains a gap in female perceived self-confidence in AI technology related terminology and tasks, there is no difference in performance or accuracy between genders (70). Kim Nilsson writes in “Forbes,” that, to mitigate service inequalities, it is essential that those professionals working in AI are representative of the population for which the AI will be used (71). There therefore, may need to be more targeted investigation into the causes for this disparity to allow timely intervention in education, training, mentorship, and representation before further integration of AI into this female-dominated clinical setting.

The Digital Natives Report (72), a multi-generational survey of over 1,000 UK business decision makers reported that AI is used in the daily lives of those born after mid-1995, so-called “Generation Z,” the youngest participants in the survey. The report also found that those in this age category have a hunger for new technology and are comfortable using it. The findings from our survey support this by the relationship found between the diagnostic radiography respondents' role and the perception of adequacy of training available in AI. The greatest discrepancy between actual and expected responses, as determined by the likelihood ratio, noted was in the student radiography cohort, with three times as many responses than predicted disagreeing with the statement “There is enough training on AI currently available for radiographers.” Additionally, there was a relationship found between role and understanding of AI (yes, no and unsure responses available). Interrogation of the responses would indicate that student therapeutic radiographers were more likely than expected, based on the likelihood ratio, to respond that they did not understand AI, and less likely to respond “yes” (Supplementary Table 12). The young professionals, and radiography students, of today are ready to embrace technology and education providers and employers should be in a position to maximise this potential.

A positive correlation between respondents' age and perceived confidence in AI and years practising and perceived confidence in AI was found in the radiotherapy responses, indicating that those in the younger age categories and those with fewer years' experience felt less confident in AI, which to some extent contradicts the literature referenced above. This may be due to progressively greater exposure to new technologies in the clinical setting over time (61). Also a positive correlation was found between confidence on AI tems and applications and highest academic degree, which suggests the need for a customised approach to AI learning provisions for different healthcare practitioners depending on the level of their prior knowledge, as expected.

Finally, a correlation was also found between diagnostic radiographers' UK region and confidence in the terminology of AI, although interrogation of the crosstabulation revealed no apparent pattern (Supplementary Table 12).

## Limitations and Future Research

This exploratory study gathered responses from a diverse sample of diagnostic and therapeutic radiographers, focussing on the UK radiography workforce. The male to female ratio (1:3) and diagnostic-to-therapeutic radiographers ratio (4:1) within the survey are representative of the actual UK radiography workforce. However, given that the survey employs convenience sampling (53), the results cannot be generalisable to the wider UK radiography population. This might relate to selection bias in relation to IT literacy and interest and knowledge of AI, as the participants were invited from the professional networks of the co-authors, many of which are established academics and researchers in the AI field. In reality the results of this work may possibly underestimate the lack of knowledge, skills, and confidence about AI as the respondents may come from settings of more established AI cultures and environments. However, convenience sampling remains an inexpensive sampling method for hard-to-reach populations (53). The sample size and sampling method is also comparable with similar studies in the field of radiography in other countries (34, 35).

Limited free response information was obtained as many of the questions required Likert-scale or closed type responses. The team is planning focus groups with purposive sampling to understand in greater depth the educational need and challenges faced with the upcoming integration of clinical AI.

The study is exploratory in nature to set the basis for future studies; hence a hypothesis was not used but an explicit aim with objectives was stated alluding to workforce readiness for AI adoption.

Finally, the survey instrument used did not employ a validated knowledge, skills, confidence scale as the team wished to contextualise and customise the survey to the priorities and needs of the workforce and validated questionnaires do not offer that flexibility; instead survey questions were developed by professional experts to get the information required to inform practise change in educational provisions in the near future.

It is hoped that this study will provide some useful material for future studies to build on.

## Conclusion

The results from this survey demonstrate that the UK radiography workforce is not yet knowledgeable, appropriately skilled, confident, or sufficiently educated for full integration of modern AI into the clinical setting. Some of the workforce are resorting to educating themselves on AI using short courses online but there is a need to prioritise formalised education and mentoring at all levels of the profession. This should not discriminate against those who do not have or do not wish to have postgraduate qualifications but also should allow flexibility by availability of postgraduate and CPD provisions for those who wish to keep abreast of technological developments after graduation. Radiographers, as integral to patient care and as direct consumers of AI technologies, need to be educated to critically embrace the emerging technologies, to ensure optimal patient care and outcomes and to be able to lead the way toward an AI-enabled future in health care.

Radiographers are usually the first and, many times, the only point of patient contact in medical imaging or radiotherapy service. Consequently, an imperative exists for all radiographers to be part of the conversation as equal members in the decision making and co-designers of any new AI technological developments in the clinical setting. In order to appropriately engage in these conversations, we need to have a workforce where all feel confident and adequately educated to be able to have a critical appreciation of the technology, its capabilities, challenges, and risks. This should come naturally for the radiography workforce, which has been traditionally trained on the interface between technological innovation and patient care. This does not mean that radiographers need to become computer science experts; but it does mean that they should be in position to safely and expertly apply AI solutions in clinical practise, be able to meaningfully appraise, interpret, and apply the evidence from literature for the benefit of their patients and collaborate in the design of new AI solutions addressing clinical challenges. With this realised, the radiographic profession would in a position to procure, use, and validate the most clinically useful AI tools for the context and patient population within which they operate, and additionally, influence the system interfaces to allow for optimal integration into current workflows.

## Data Availability Statement

The original contributions presented in the study are included in the article/Supplementary Material, further inquiries can be directed to the corresponding author.

## Ethics Statement

The studies involving human participants were reviewed and approved by City, University of London SHS REC. The patients/participants provided their written informed consent to participate in this study. Electronic consenting format was used in this online survey.

## Author Contributions

CM and SM have equally contributed to the conceptualisation and design of this study and are therefore sharing joint last authorship. CR, CM, SM, and TO'R have contributed to different aspects of data analysis and write up. All authors contributed to the design of the online survey, recruitment of study participants, reviewed different drafts of this document, and approved the final draft.

## Funding

We would like to thank the City University Radiography Research Fund 90020HY for covering the costs of dissemination for this publication.

## Conflict of Interest

The authors declare that the research was conducted in the absence of any commercial or financial relationships that could be construed as a potential conflict of interest.

## Publisher's Note

All claims expressed in this article are solely those of the authors and do not necessarily represent those of their affiliated organizations, or those of the publisher, the editors and the reviewers. Any product that may be evaluated in this article, or claim that may be made by its manufacturer, is not guaranteed or endorsed by the publisher.

## References

[1] Shen D Wu G Suk H-I. Deep learning in medical image analysis. Annu Rev Biomed Eng. (2017) 19:221–48. 10.1146/annurev-bioeng-071516-044442 28301734 PMC5479722

[2] Erickson BJ. Ch 4: Deep learning and machine learning in imaging: basic principles. In: Ranschaert ER Morozov S Algra PR, editors. Artificial Intelligence in Medical Imaging. Cham: Springer Nature Switzerland (2019). p. 39–46. 10.1007/978-3-319-94878-2_4

[3] Meijering M. A bird's-eye view of deep learning in bioimage analysis. Comput Struct Biotechnol J. (2020) 18:2312–25. 10.1016/j.csbj.2020.08.003 32994890 PMC7494605

[4] England JR Cheng PM. Artificial intelligence for medical image analysis: a guide for authors and reviewers. Am J Radiol. (2019) 212:513–9. 10.2214/AJR.18.20490 30557049

[5] Huisman M Ranschaert E Parker W Mastrodicasa D Koci M Pinto de. Santos D, et al. An international survey on AI in radiology in 1,041 radiologists and radiology residents part 1: fear of replacement, knowledge, and attitude. Eur Radiol. (2021) 31:7058–66. 10.1007/s00330-021-07781-5 33744991 PMC8379099

[6] Recht M Bryan M. Artificial intelligence: threat or boon to radiologists? J Amer Coll Radiol. (2017) 14:11. 10.1016/j.jacr.2017.07.007 28826960

[7] Chockley K Emanuel E. The end of radiology? Three threats to the future practice of radiology. J Amer Coll Radiol. (2016) 13:1415–20. 10.1016/j.jacr.2016.07.010 27652572

[8] *NHS Long Term Plan* (2019). Available online at: https://www.longtermplan.nhs.uk/ (accessed November 24, 2020).

[9] Waymel Q Badr S Demondion X Cotten A Jacques T. Impact of the rise of artificial intelligence in radiology: what do radiologists think? Diagn Interv Imaging. (2019) 100:327–36. 10.1016/j.diii.2019.03.015 31072803

[10] Oh S Kim JH Choi SW Lee HJ Hong J Kwon SH. Physician confidence in artificial intelligence: an online mobile survey. J Med Internet Res. (2019) 21:e12422. 10.2196/12422 30907742 PMC6452288

[11] Pinto Dos Santos D Giese D Brodehl S Chon SH Staab W Kleinert R. Medical students' attitude towards artificial intelligence: a multicentre survey. Eur Radiol. (2019) 29:1640–6. 10.1007/s00330-018-5601-1 29980928

[12] Abdullah R Fakieh B. Health care employees' perceptions of the use of artificial intelligence applications: survey study. J Med Internet Res. (2020) 22:e17620. 10.2196/17620 32406857 PMC7256754

[13] Park CJ Yi PH Siegel EL. Medical student perspectives on the impact of artificial intelligence on the practice of medicine. Curr Probl Diagn Radiol. (2020). 50:614–9. 10.1067/j.cpradiol.2020.06.011 32680632

[14] Philpotts L. Can computer-aided detection be detrimental to mammographic interpretation? Radiology. (2009) 253:17–22. 10.1148/radiol.2531090689 19789251

[15] Kitamura FC Marques O. Trustworthiness of artificial intelligence models in radiology and the role of explainability. Amer Coll Radiol. (2021) 8:1160–2. 10.1016/j.jacr.2021.02.008 33676912

[16] Schuur F Mehrizi MHR Ranschaert E. Training opportunities of artificial intelligence (AI) in radiology: a systemic review. Eur Radiol. (2021) 31:6021–29. 10.1007/s00330-020-07621-y 33587154 PMC8270863

[17] Kelly CJ Karthikesalingam A Suleyman M Corrado G King D. Key challenges for delivering clinical impact with artificial intelligence. BMC Med. (2019) 17:195. 10.1186/s12916-019-1426-2 31665002 PMC6821018

[18] Nagendran M Chen Y Lovejoy CA Gordon AC Komorowski M Harvey H. Artificial intelligence versus clinicians: systematic review of design, reporting standards, and claims of deep learning studies. Brit Med J (2020) 368:m689. 10.1136/bmj.m689 32213531 PMC7190037

[19] Sit C Srinivasan R Amlani A Muthuswamy K Azam A Monzon L. Attitudes and perceptions of UK medical students towards artificial intelligence and radiology: a multicentre survey. Insights Imaging. (2020) 11:14. 10.1186/s13244-019-0830-7 32025951 PMC7002761

[20] RCR New RCR Census Shows The NHS Needs Nearly 2 000 More Radiologists (2021). Available online at: https://www.rcr.ac.uk/posts/new-rcr-census-shows-nhs-needs-nearly-2000-more-radiologists (accessed September 1, 2021).

[21] Society and College of Radiographers. Radiography Census Highlights Staff Bravery Amid Workforce Shortages. Available online at: Radiography census highlights staff bravery amid workforce shortages | SoR (accessed September 1, 2021).

[22] The Society of Radiographers 2020 Annual Report A Century of Success. London: Society of Radiographers (2020). Available online at: GetFile.aspx (https://www.sor.orgsor.org) (accessed July 8, 2021).

[23] HCPC. Registrant Snapshot (2021). Available online at: https://www.hcpc-uk.org/about-us/insights-and-data/the-register/registrant-snapshot-may-2021/ (accessed: June 25, 2021).

[24] Liew C. The future of radiology segmented with artificial intelligence: a strategy for success. Eur J Radiol. (2018) 102:152–6. 10.1016/j.ejrad.2018.03.019 29685530

[25] Hardy MA Harvey H. Artificial intelligence in diagnostic imaging: impact on the radiography profession. Br J Radiol. (2020) 93:20190840. 10.1259/bjr.20190840 31821024 PMC7362930

[26] Duan Y Edwards JS Dwivedi Y. Artificial intelligence for decision making in the era of Big Data – evolution, challenges and research agenda. Int J Inform Manage. (2019) 48. 10.1016/j.ijinfomgt.2019.01.021

[27] Chang A. Intelligence Based Medicine. London: Academic Press. (2020).

[28] Castellino RA. Computer aided detection (CAD): an overview. Cancer Imaging. (2005) 5:17–9. 10.1102/1470-7330.2005.0018 16154813 PMC1665219

[29] Fazal MI Patel ME Tye J Gupta Y. The past, present and future role of artificial intelligence in imaging. Eur J Radiol. (2018) 105:246–50. 10.1016/j.ejrad.2018.06.020 30017288

[30] Langlotz CP Allen B Erickson BJ Kalpathy-Cramer J Bigelow K Cook TS. A roadmap for foundational research on artificial intelligence in medical imaging: from the 2018. NIH/RSNA/AC/The Academy Workshop. Radiology. (2019) 291:190613. 10.1148/radiol.2019190613 30990384 PMC6542624

[31] Chen Y Stavropoulou C Narasinkan R Baker A Scarbrough H. Professionals' responses to the introduction of AI innovations in radiology and their implications for future adoption: a qualitative study. BMC Health Serv Res. (2021) 21:813. 10.1186/s12913-021-06861-y 34389014 PMC8364018

[32] Wong K Gallant F Szumacher E. Perceptions of Canadian radiation oncologists, radiation physicists, radiation therapists and radiation trainees about the impact of AI in Radiation Oncology. J Med Imag Radiat Sci. (2021) 52:44e8. 10.1016/j.jmir.2020.11.013 33323332

[33] American Society of Radiologic Technologists. 2019 Artificial Intelligence Survey. American Society of Radiologic Technologists (2019). Available online at: https://www.asrt.org/docs/default-source/research/2019-artificial-intelligence-survey.pdf?sfvrsnij95033fd0_4survey (accessed June 10, 2021).

[34] Ryan ML O'Donovan T McNulty JP. Artificial intelligence: the opinions of radiographers and radiation therapists in Ireland. Radiography (2021) 27(suppl. 1):74–82. 10.1016/j.radi.2021.07.022 34454835

[35] Abuzaid MM Elshami W Tekin H Issa B. Assessment of the willingness of radiologists and radiographers to accept the integration if artificial intelligence into radiology practice. Acad Radiol. (2020) 2020:S1076-6332(20)30553-5. 10.1016/j.acra.2020.09.014 33129659

[36] Botwe BO Antwi WK Arkoh S Akudjedu TN. Radiographers' perspectives on the emerging integration of artificial intelligence into diagnostic imaging: the Ghana study. J Med Radiat Sci. (2021) 68:260–8. 10.1002/jmrs.460 33586361 PMC8424310

[37] Sarwar S Dent A Faust K Richer M Djuric U Van Ommeren R. Physician perspectives on integration of artificial intelligence into diagnostic pathology NPJ Digit Med. (2019) 2:28. 10.1038/s41746-019-0106-0 31304375 PMC6550202

[38] Kumar D Wong A Taylor GW. Explaining the Unexplained: A Class-Enhanced Attentive Response (CLEAR) Approach to Understanding Deep Neural Networks (2018). Available online at: https://ieeexplore.ieee.org/Xplore/home.jsp (accessed August 10, 2019). 10.1109/CVPRW.2017.215

[39] Reyes M Meier R Pereira S Silva CA Dahlweid F-M von Tengg-Kobligk H. On the interpretability of artificial intelligence in radiology: challenges and opportunities. Radiol Artif Intell. (2020) 2:e190043. 10.1148/ryai.2020190043 32510054 PMC7259808

[40] NHS The Topol Review. Health Education England (2019). Available online at: https://topol.hee.nhs.uk/ (accessed May 5, 2021).

[41] HCPC. Proposed changes to the HCPC Standards of Proficiency (Radiographers) (2020). Available online at: https://www.hcpc-uk.org/globalassets/consultations/2020/standards-of-proficiency/radiographers/table-of-proposed-changes—radiographers.pdf (accessed June 23, 2020).

[42] International Society of Radiographers and Radiological Technologists and the European Federation of Radiographer Societies. Artificial intelligence and the radiographer/radiological technologist profession: a joint statement of the International Society of Radiographers and Radiological Technologists and the European Federation of Radiographer Societies. Radiography. (2020) 26, 93–5. 10.1016/j.radi.2020.03.007 32252972

[43] Malamateniou C McFadden S McQuinlan Y England A Woznitza N Goldsworthy S. Artificial intelligence: guidance for clinical imaging and therapeutic radiography professionals, a summary by the Society of Radiographers AI working group. Radiography. (2021). 27:1192–202. 10.1016/j.radi.2021.07.028 34420888

[44] Laï MC Brian M Mamzer MF. Perceptions of artificial intelligence in healthcare: findings from a qualitative survey study among actors in France. J Transl Med. (2020) 18:14. 10.1186/s12967-019-02204-y 31918710 PMC6953249

[45] Evans JR Mathur A. The value of online surveys. Internet Res. (2018) 28:4. 10.1108/IntR-03-2018-0089

[46] Eysenbach G. Improving the quality of web surveys: the Checklist for Reporting Results of Internet E-Surveys (CHERRIES). J Med Internet Res. (2004) 6:e34. 10.2196/jmir.6.3.e34 15471760 PMC1550605

[47] Tavakol M Dennick R. Making sense of Cronbach's alpha. Int J Med Educ. (2011) 27:53–5. 10.5116/ijme.4dfb.8dfd 28029643 PMC4205511

[48] Streiner DL Norman GR Cairney J. Health Measurement Scales: A Practical Guide to their Development and Use. 5th ed. Oxford: Oxford University Press (2015).

[49] Oluwatayo J. Validity and reliability issues in educational research. J Educ Soc Res. (2012) 2:391–400. Available online at: https://www.richtmann.org/journal/index.php/jesr/article/view/11851

[50] Straub D Boudreau M Gefen D. Validation guidelines for IS positivist research. Commun Assoc Inform Syst. (2004) 13:380–427. 10.17705/1CAIS.01324

[51] Baltar F Brunet I. Social research 20: virtual snowball sampling method using Facebook. Internet Res. (2012) 22:57–74. 10.1108/10662241211199960

[52] *NVivo Qualitative Data Analysis Software; QSR International Pty Ltd*. Version 12 (2018).

[53] Fricker RD Jr. Chapter 10: Sampling methods for online surveys. In: Fielding N Lee RM Blank G, editors. The SAGE Handbook of Online Research Methods. 2nd ed. London: SAGE Publications (2017) 162–83. 10.4135/9781473957992.n10

[54] Pallant J. SPSS Survival Manual. 3rd ed. Berkshire: Open University Press/McGraw-Hill (2007).

[55] *IBM SPSS Statistical Package for Windows Version 23*. Armonk, NY: IBM Corporation (2019).

[56] Field A. Discovering Statistics Using IBM SPSS Statistics. 4th ed. Sage: London (2013).

[57] HCPC. Number of therapeutic radiographers on the HCPC Register (2018). Available online at: https://www.hcpc-uk.org/resources/freedom-of-information-requests/2018/number-of-therapeutic-radiographers-on-the-hcpc-register—may-2018/ (accessed June 15, 2021).

[58] Tejani AS. Identifying and addressing barriers to an artificial intelligence curriculum. Amer Coll Radiol. (2020) 18:4. 10.1016/j.jacr.2020.10.001 33823981

[59] *SIIM Strategic Plan 2017–2020* (2017). Available online at: https://cdn.ymaws.com/siim.org/resource/resmgr/governance/strategic_plan_2017v22.pdf (accessed June 16, 2021).

[60] Lindqwister AL Hassanpour S Lewis PJ Sin JM. AI-RADS: an artificial intelligence curriculum for residents. Acad Radiol. (2020) 20:1076–6332. 10.1016/j.acra.2020.09.017 33071185 PMC7563580

[61] Hafslund B Clare J Graverholt B. Wammen-Nortvedt, M. Evidence-based radiography. Radiography. (2008) 14:4. 10.1016/j.radi.2008.01.003

[62] Aarts S Cornelis F Zevenboom Y Brokken P van de Griend N Spoorenberg M. The opinions of radiographers, nuclear medicine technologists and radiation therapists regarding technology in healthcare: a qualitative study. J Med Radiat Sci. (2017) 64:3–9. 10.1002/jmrs.207 28303693 PMC5355371

[63] SECTRA: The Radiologist's Handbook for Future Excellence (2021). Available online at: https://medical.sectra.com/resources/the-radiologists-handbook-for-future-excellence-2021/ (accessed June 15, 2021).

[64] Dunning D. The Dunning-Kruger effect: on being ignorant of one's own ignorance. In: Olsen JM Zanna MP, editors. Advances in Experimental Social Psychology, Vol. 44. Cambridge, MA: Academic Press (2011), 247–96. 10.1016/B978-0-12-385522-0.00005-6

[65] Ehrlinger J Dunning D. How chronic self-views influence (and potentially mislead) estimates of performance. J Pers Soc Psychol. (2003) 84:1. 10.1037/0022-3514.84.1.5 12518967

[66] Knowles MS. Andragogy in Action. Applying Modern Principles of Adult Education. San Francisco, CA: Jossey Bass (1984).

[67] West SM Whittaker M Crawford K. Discriminating Systems: Gender, Race and Power in AI. AI Now Institute (2019). Available online at: https://ainowinstitute.org/discriminatingsystems.html (accessed June 16, 2021).

[68] Yau HK Cheng ALF. Gender difference of confidence in using technologyfor learning. J Technol Stud. (2012) 38:74–9. 10.21061/jots.v38i2.a.2

[69] Royal College of Radiologists. Clinical Radiology UK Workforce Census (2020). Available online at: https://www.rcr.ac.uk/system/files/publication/field_publication_files/clinical-radiology-uk-workforce-census-2020-report.pdf (accessed June 15, 2021).

[70] Liberatore MJ Wagner WP. Gender, performance, and self-efficacy: a quasi-experimental field study. J Comput Inform Syst. (2020). 10.1080/08874417.2020.1717397

[71] Nilsson K. Why AI needs more women. Forbes (2019). Available online at: https://www.forbes.com/sites/kimnilsson/2019/03/08/why-ai-needs-more-women/?sh=13a953577f90 (accessed June 19, 2021).

[72] Advanced. The Digital Natives Report (2019). Available online at: https://www.oneadvanced.com/trends-report/digital-natives-report-2019-2020/ (accessed June 29, 2021).

