# Supplementary material for: Beauty Is in the AI of the Beholder: Are We Ready for the Clinical Integration of Artificial Intelligence in Radiography? An Exploratory Analysis of Perceived AI Knowledge, Skills, Confidence, and Education Perspectives of UK Radiographers
Source: Front Digit Health. 2021 Nov 11;3:739327. doi: 10.3389/fdgth.2021.739327 (PMC8631824; doi:10.3389/fdgth.2021.739327)

Supplementary Table 1 Coding of qualitative data using thematic analysis

(please refer also to Supplementary Figures 1 and 2)

| **THEMES** | **CODES** |
| --- | --- |
| **Clinical applications of AI** | Anatomy, pathology, identification of pathology or diagnosis  Tasks which normally require human input / human like qualities  Decision making problem solving  Segmentation, planning, contouring  Speech recognition    Education and simulation  Image evaluation and quality – quality control, image acquisition  Triage workflow management |
| **Advantages of AI** | Benefits to clinician  Speed efficiency  Benefits to patient  Accuracy  Service improvements  Reliable and trustworthy  Improve outcomes |
| **Disadvantages of AI** | Disadvantages  Displacing or replacing jobs |
| **Technical description of the technology with data science terms.** | Modern AI technology  Old AI -pattern recognition  Robots  Computers thinking for themselves |

Supplementary table 2: ***Ordinal v ordinal correlation (Spearman’s rank and Kendall’s tau)***

| **INDEPENDENT** | **DEPENDENT** | **Diagnostic Radiography**  (Kendall’s tau and Spearman’s rank respectively) | **Radiotherapy**  (Kendall’s tau and Spearman’s rank respectively)  *P=two tailed* |
| --- | --- | --- | --- |
| **Age** | *Understanding of AI (Scale 0-10)* | Not significant  *P=0.65; 0.61* | 0.313; 0.417, significant positive correlation at 0.01 level *(p=0.01; p<0.00)*  Moderate +ve correlation |
|  | *Confidence using AI technologies (4-point Likert)* | Not significant  *P=0.614; 0.587* | Not significant  *P=0.211; 0.211* |
|  | *Confidence in AI terminology (4-point Likert)* | Not significant  *P=0.988; 0.998* | Not significant  *p=0.287; 0.296* |
|  | *Perception of adequacy of level of training (7-point Likert)* | Not significant  *P=0.306; 0.305* | Not significant  *p=0.563; 0.565* |
|  | *Perception of having developed/learned some skill in AI (7-point Likert)* | Not significant  *P=0.773; 0.778* | Not significant  *p=0.255; 0.254* |
|  | *Opinion on availability of training (7-point Likert)* | Not significant  *P=0.243; 0.266* | Not significant  *p=0.861; 0.924* |
| **Years’ experience** | *Understanding of AI (Scale 0-10)* | Not significant  *P=0.150; 0.144* | 0.332; 0.437, significant at 0.01 level *(p<0.00; p<0.00)*  Moderate +ve correlation |
|  | *Confidence using AI technologies (4-point Likert)* | Not significant  *P=0.674; 0.684* | Not significant  *p=0.389; 0.382* |
|  | *Confidence in AI terminology (4-point Likert)* | Not significant  *P=0.476; 0.478* | Not significant  *p=0.870; 0.890* |
|  | *Perception of adequacy of level of training (7-point Likert)* | Not significant  *P=0.600; 0.600* | Not significant  *p=0.808; 0.778* |
|  | *Perception of having developed/learned some skill in AI (7-point Likert)* | Not significant  *P=0.270; 0.275* | Not significant  *p=0.388; 0.379* |
|  | *Opinion on availability of training (7-point Likert)* | Not significant  *P=0.927; 0.893* | Not significant  *p=0.810; 0.841* |
| **Level of highest academic qualification** | *Understanding of AI (Scale 0-10)* | 0.201; 0.258, significant at 0.01 level *(p<0.00; p<0.00)*  Moderate +ve correlation | 0.355; 0.429, significant at 0.01 level *(p<0.00; p<0.00)*  Moderate +ve correlation |
|  | *Confidence using AI technologies (4-point Likert)* | Not significant  *P=0.077; 0.080* | Not significant  *p=0.174; 0.167* |
|  | *Confidence in AI terminology (4-point Likert)* | 0.218; 0.151, significant at 0.05 level *(p = 0.05)*  +ve correlation | Not significant  *p=0.298; 0.304* |
|  | *Perception of adequacy of level of training (7-point Likert)* | Not significant  *P=0.141; 0.146* | Not significant  *p=0.592; 0.613* |
|  | *Perception of having developed/learned some skill in AI (7-point Likert)* | Not significant  *P=0.098; 0.098* | Not significant  *p=0.098; 0.098* |
|  | *Opinion on availability of training (7-point Likert)* | Not significant  *P=0.783; 0.789* | Not significant  *p=0.783; 0.789* |

***Supplementary Table 3: Sub group analysis of DR highest academic qualification and understanding of AI (scale 0-10***

| **DR What is your highest academic qualification? - Selected Choice * DR On a scale of 0-10 : understand 'artificial intelligence' Counts/total responses (proportion)** | | | | | | | | | | | | | | | |
| --- | --- | --- | --- | --- | --- | --- | --- | --- | --- | --- | --- | --- | --- | --- | --- |
|  | | | | | | | | | | | | | | | |
|  | | DR On a scale of 0-10 : understand 'artificial intelligence' | | | | | | | | | | | | Total | |
|  |  | .00 | 1.00 | 2.00 | 3.00 | 4.00 | 5.00 | 6.00 | 7.00 | 8.00 | 9.00 | 10.00 |  | |  |
| DR What is your highest academic qualification? - Selected Choice | A-level or equivalent | 4/39 | 1/39 | 8/39 | 2/39 | 4/39 | 5/39 | 5/39 | 5/39 | 3/39 | 1/39 | 1/39 | 39 | |  |
|  | BSc | 1/70 | 6/70 | 7/70 | 4/70 | 8/70 | 7/70 | 10/70 | 15/70 | 7/70 | 3/70 | 2/70 | 70 | |  |
|  | Pg Certificate | 3/61 | 3/61 | 6/61 | 5/61 | 4/61 | 6/61 | 12/61 | 14/61 | 3/61 | 3/61 | 2/61 | 61 | |  |
|  | Pg Diploma | 2/38 | 2/38 | 3/38 | 2/38 | 1/38 | 5/38 | 6/38 | 10/38 | 6/38 | 1/38 | 0/38 | 38 | |  |
|  | MSc | 0/57 | 0/57 | 1/57 | 2/57 | 3/57 | 8/57 | 12/57 | 13/57 | 11/57 | 3/57 | 4/57 | 57 | |  |
|  | PhD/EdD/DProf or equivalent | 0/6 | 0/6 | 0/6 | 0/6 | 0/6 | 1/6 | 0/6 | 3/6 | 1/6 | 1/6 | 0/6 | 6 | |  |
|  | Other. Please clarify below: | 1/20 | 0/20 | 1/20 | 0/20 | 1/20 | 3/20 | 3/20 | 6/20 | 2/20 | 1/20 | 2/20 | 20 | |  |
| Total | | 11 | 12 | 26 | 15 | 21 | 35 | 48 | 66 | 33 | 13 | 11 | 291 | |  |

***Supplementary Table 4***

***Sub group analysis of DR highest academic qualification and understanding of AI (scale 0-10) with grouping of low (0-3), medium (4-6) and high understanding (7-10)]***

|  | ***DR - ‘On a scale of 0 – 10, how well do you understand the term ‘Artificial Intelligence’?’***  ***Counts / total responses (proportion)*** | | |
| --- | --- | --- | --- |
| ***‘What is your highest academic qualification?’*** | ***0 – 3*** | ***4 – 6*** | ***7 – 10*** |
| A-level or equivalent | 15/39 (0.38) | 14/39 (0.36) | 10/39 (0.26) |
| BSc | 18/70 (0.26) | 25/70 (0.36) | 27/70 (0.39) |
| Pg Certificate | 17/61 (0.28) | 22/61 (0.36) | 22/61 (0.36) |
| Pg Diploma | 9/38 (0.24) | 12/38 (0.32) | 17/38 (0.45) |
| MSc | 3/57 (0.05) | 23/57 (0.40) | 31/57 (0.54) |
| PhD/EdD/DProf or equivalent | 0/6 (0) | 1/6 (0.17) | 5/6 (0.83) |
| Other | 2/20 (0.10) | 7/20 (0.35) | 11/20 (0.55) |

***Supplementary Table 5***

***Sub group analysis of RT highest academic qualification, understanding of AI (scale 0-10)***

| **RT What is your highest academic qualification? * RT On a scale of 0-10 : understand 'artificial intelligence' (counts)** | | | | | | | | | | | | | | | |
| --- | --- | --- | --- | --- | --- | --- | --- | --- | --- | --- | --- | --- | --- | --- | --- |
|  | | | | | | | | | | | | | | | |
|  | | RT On a scale of 0-10 : understand 'artificial intelligence' | | | | | | | | | | | | Total | |
|  |  | .00 | 1.00 | 2.00 | 3.00 | 4.00 | 5.00 | 6.00 | 7.00 | 8.00 | 9.00 | 10.00 |  | |  |
| RT What is your highest academic qualification? | A-level or equivalent | 2 | 1 | 0 | 2 | 0 | 2 | 0 | 0 | 0 | 0 | 0 | 7 | |  |
|  | BSc | 2 | 1 | 4 | 8 | 3 | 3 | 1 | 1 | 2 | 1 | 0 | 26 | |  |
|  | Pg Certificate | 0 | 0 | 0 | 0 | 0 | 0 | 1 | 0 | 0 | 0 | 0 | 1 | |  |
|  | Pg Diploma | 0 | 0 | 0 | 0 | 0 | 1 | 3 | 1 | 0 | 0 | 0 | 5 | |  |
|  | MSc | 1 | 0 | 2 | 4 | 2 | 2 | 7 | 6 | 1 | 0 | 1 | 26 | |  |
|  | PhD/EdD/DProf or equivalent | 0 | 0 | 0 | 0 | 1 | 0 | 0 | 1 | 1 | 0 | 0 | 3 | |  |
|  | Other. Please clarify below: | 0 | 0 | 0 | 2 | 1 | 0 | 0 | 0 | 0 | 0 | 0 | 3 | |  |
| Total | | 5 | 2 | 6 | 16 | 7 | 8 | 12 | 9 | 4 | 1 | 1 | 71 | |  |

***Supplementary Table 6***

***Sub group analysis DR highest academic qualification, understanding of AI (scale 0-10),***

***[with grouping of low (0-3), medium (4-6) and high understanding (7-10)]***

|  | ***RT - ‘On a scale of 0 – 10, how well do you understand the term ‘Artificial Intelligence’?’*** | | |
| --- | --- | --- | --- |
| ***‘What is your highest academic qualification?’*** | ***0 – 3***  ***counts / total responses (proportion)*** | ***4 – 6*** | ***7 – 10*** |
| A-level or equivalent | 5/7 (0.71) | 2/7 (0.29) | 0/7 (0) |
| BSc | 15/26 (0.57) | 7/26 (0.27) | 4/26 (0.15) |
| Pg Certificate | 0/1 (0) | 1/1 (1) | 0/1 (0) |
| Pg Diploma | 0/5 (0) | 4/5 (0.8) | 1/5 (0.2) |
| MSc | 7/26 (0.27) | 11/26 (0.42) | 8/26 (0.31) |
| PhD/EdD/DProf or equivalent | 0/3 (0) | 1/3 (0.33) | 2/3 (0.67) |
| Other | 2/3 (0.67) | 1/3 (0.33) | 0/3 (0) |

***Supplementary Table 7***

***Sub group analysis of DR highest academic qualification, confidence in the terminology of AI***

| **DR What is your highest academic qualification? - Selected Choice * DR Do you feel confident in your understanding of the underlying terminology of AI. (counts)** | | | | | | |
| --- | --- | --- | --- | --- | --- | --- |
|  | | | | | | |
|  | | DR Do you feel confident in your understanding of the underlying terminology of AI. | | | | Total |
|  |  | Not confident at all | Somewhat confident | Confident enough | Very confident |  |
| DR What is your highest academic qualification? - Selected Choice | A-level or equivalent | 21/39 (0.51) | 11/39 (0.28) | 6/39 (0.15) | 1/39 (0.03) | 39 |
|  | BSc | 29/70 (0.41) | 29/70 (0.41) | 10/70 (0.14) | 2/70 (0.03) | 70 |
|  | Pg Certificate | 33/61 (0.54) | 17/61 (0.28) | 10/61 (0.16) | 1/61 (0.02) | 61 |
|  | Pg Diploma | 16/38 (0.42) | 17/38 (0.45) | 4/38 (0.11) | 1/38 (0.03) | 38 |
|  | MSc | 15/57 (0.26) | 24/57 (0.42) | 15/57 (0.26) | 3/57 (0.05) | 57 |
|  | PhD/EdD/DProf or equivalent | 1/6 (0.17) | 4/6 (0.67) | 1/6 (0.17) | 0/6 (0) | 6 |
|  | Other. Please clarify below: | 8/20 (0.4) | 9/20 (0.45) | 3/20 (0.15) | 0/20 (0) | 20 |
| Total | | 123 | 111 | 49 | 8 | 291 |

***Supplementary Table 8:*** ***Sub group analysis RT Age, understanding of AI (scale 0-10)***

| **RT What is your age range? * RT On a scale of 0-10 : understand 'artificial intelligence' (counts)** | | | | | | | | | | | | | | | |
| --- | --- | --- | --- | --- | --- | --- | --- | --- | --- | --- | --- | --- | --- | --- | --- |
|  | | | | | | | | | | | | | | | |
|  | | RT On a scale of 0-10 : understand 'artificial intelligence' | | | | | | | | | | | | Total | |
|  |  | .00 | 1.00 | 2.00 | 3.00 | 4.00 | 5.00 | 6.00 | 7.00 | 8.00 | 9.00 | 10.00 |  | |  |
| RT What is your age range? | 18-25 years old | 2 | 2 | 3 | 5 | 1 | 2 | 1 | 0 | 0 | 0 | 0 | 16 | |  |
|  | 26-35 years old | 2 | 0 | 2 | 6 | 2 | 2 | 2 | 2 | 1 | 0 | 1 | 20 | |  |
|  | 36-45 years old | 0 | 0 | 1 | 2 | 2 | 1 | 2 | 5 | 2 | 1 | 0 | 16 | |  |
|  | 46-55 years old | 1 | 0 | 0 | 1 | 1 | 3 | 6 | 2 | 0 | 0 | 0 | 14 | |  |
|  | 55-65 years old | 0 | 0 | 0 | 2 | 1 | 0 | 1 | 0 | 1 | 0 | 0 | 5 | |  |
| Total | | 5 | 2 | 6 | 16 | 7 | 8 | 12 | 9 | 4 | 1 | 1 | 71 | |  |

***Supplementary Table 9***

***Sub group analysis RT age, understanding of AI (scale 0-10), with grouping of low (0-3), medium (4-6) and high understanding (7-10)***

|  | ***RT - On a scale of 0 – 10, how well do you understand the term ‘Artificial Intelligence’?***  ***Counts / total responses (proportion)*** | | |
| --- | --- | --- | --- |
| ***What is your age range?*** | ***0 – 3*** | ***4 – 6*** | ***7 – 10*** |
| 18-25 years old | 12/16 (0.75) | 4/16 (0.25) | 0/16 (0) |
| 26-35 years old | 10/20 (0.5) | 6/20 (0.3) | 4/20 (0.2) |
| 36-45 years old | 3/16 (0.19) | 5/16 (0.31) | 8/16 (0.5) |
| 46-55 years old | 2/14 (0.14) | 10/14 (0.71) | 2/14 (0.14) |
| 55-65 years old | 2/5 (0.40) | 2/5 (0.40) | 1/5 (0.20) |

***Supplementary Table 10: Sub-group analysis RT Year practicing, understanding of AI (scale 0-10)***

| **RT How many years have you practiced radiotherapy * RT On a scale of 0-10 : understand 'artificial intelligence' (counts)** | | | | | | | | | | | | | | | |
| --- | --- | --- | --- | --- | --- | --- | --- | --- | --- | --- | --- | --- | --- | --- | --- |
|  | | | | | | | | | | | | | | | |
|  | | RT On a scale of 0-10 : understand 'artificial intelligence' | | | | | | | | | | | | Total | |
|  |  | .00 | 1.00 | 2.00 | 3.00 | 4.00 | 5.00 | 6.00 | 7.00 | 8.00 | 9.00 | 10.00 |  | |  |
| RT How many years have you practiced radiotherapy | 0-2 years | 3 | 1 | 2 | 4 | 1 | 3 | 1 | 1 | 0 | 0 | 0 | 16 | |  |
|  | 3-5 years | 1 | 1 | 2 | 5 | 1 | 1 | 0 | 1 | 0 | 0 | 1 | 13 | |  |
|  | 6-10 years | 0 | 0 | 2 | 3 | 1 | 0 | 1 | 1 | 1 | 0 | 0 | 9 | |  |
|  | 11-20 years | 0 | 0 | 0 | 2 | 2 | 1 | 3 | 4 | 2 | 1 | 0 | 15 | |  |
|  | >20 years | 1 | 0 | 0 | 2 | 1 | 3 | 7 | 2 | 1 | 0 | 0 | 17 | |  |
|  | I have worked as a radiographer, but I am currently in retirement | 0 | 0 | 0 | 0 | 1 | 0 | 0 | 0 | 0 | 0 | 0 | 1 | |  |
| Total | | 5 | 2 | 6 | 16 | 7 | 8 | 12 | 9 | 4 | 1 | 1 | 71 | |  |

***Supplementary Table 11***

|  | ***RT - ‘On a scale of 0 – 10, how well do you understand the term ‘Artificial Intelligence’?’*** | | |
| --- | --- | --- | --- |
| ***‘How many years have you practiced radiotherapy?’*** | ***0 – 3***  ***counts / total responses (proportion)*** | ***4 – 6*** | ***7 – 10*** |
| 0-2 years | 10/16 (0.62) | 5/16 (0.31) | 1/16 (0.07) |
| 3-5 years | 9/13 (0.69) | 2/13 (0.15) | 2/13 (0.15) |
| 6-10 years | 5/9 (0.56) | 2/9 (0.22) | 2/9 (0.22) |
| 11-20 years | 2/15 (0.13) | 6/15 (0.40) | 7/15 (0.47) |
| >20 years | 3/17 (0.17) | 11/17 (0.65) | 2/17 (0.18) |
| I have worked as a radiographer, but I am currently in retirement | 0/1 (0) | 1/1 (1) | 0/1 (0) |

***Sub-group RT Years practicing, understanding of AI (scale 0-10) with grouping of low (0-3), medium (4-6) and high understanding (7-10)***

***Supplementary Table 12: Nominal v nominal correlations (Likelihood Chi square)***

| **INDEPENDENT** | **DEPENDENT**  **DIAG RAD** | **Likelihood ratio chi-square (p-value) sig/not sig** | **Cramer’s V**  **Magnitude** | **DEPENDENT**  **RADIOTHERAPY** | **Likelihood ratio chi-square (p-value) sig/not sig** | **Cramer’s V**  **Magnitude** |
| --- | --- | --- | --- | --- | --- | --- |
| **Gender**  **V (four categories):**  Small = 0.06  Medium = 0.17  Large = 0.29 | *Understanding of AI (yes/no/unsure)* | Not sig  *p>0.05* |  | *Understanding of AI (yes/no/unsure)* | Significant p=0.020  N=71 | 0.318 (large) |
|  | *Confidence using AI technologies (4-point Likert)* | Significant p=0.000  N=291 | 0.191 (medium) | *Confidence using AI technologies (4-point Likert)* | Not sig  *p>0.05* |  |
|  | *Confidence in AI terminology (4-point Likert)* | Significant p=0.000  n=291 | 0.252 (medium) | *Confidence in AI terminology (4-point Likert)* | Significant  p=0.006  n=70 | V=0.445 (large) |
|  | *Perception of adequacy of level of training (7-point Likert)* | Not sig  *p>0.05* |  | *Perception of adequacy of level of training (7-point Likert)* | Not sig  *p>0.05* |  |
|  | *Perception of having developed/learned some skill in AI (7-point Likert)* | Not sig  *p>0.05* |  | *Perception of having developed/learned some skill in AI (7-point Likert)* | Not sig  *p>0.05* |  |
|  | *Opinion on availability of training (7-point Likert)* | Not sig  *p>0.05* |  | *Opinion on availability of training (7-point Likert)* | Not sig  *p>0.05* |  |
| **Role** | *Understanding of AI (yes/no/unsure)* | Not sig  *p>0.05* |  | *Understanding of AI (yes/no/unsure)* | Significant p=0.045, n=71 | 0.450 (large) |
|  | *Confidence using AI technologies (4-point Likert)* | Not sig  *p>0.05* |  | *Confidence using AI technologies (4-point Likert)* | Not sig  *p>0.05* |  |
|  | *Confidence in AI terminology (4-point Likert)* | Not sig  *p>0.05* |  | *Confidence in AI terminology (4-point Likert)* | Not sig  *p>0.05* |  |
|  | *Perception of adequacy of level of training (feeling of being well trained to implement new AI) (7-point Likert)* | Significant  p=0.040  n=285 | V=0.268 (medium) | *Perception of adequacy of level of training (7-point Likert)* | Not sig  *p>0.05* |  |
|  | *Perception of having developed/learned some skill in AI (7-point Likert)* | Not sig  *p>0.05* |  | *Perception of having developed/learned some skill in AI (7-point Likert)* | Not sig  *p>0.05* |  |
|  | *Opinion on availability of training (7-point Likert)* | Not sig  *p>0.05* |  | *Opinion on availability of training (7-point Likert)* | Not sig  *p>0.05* |  |
| **UK region** | *Understanding of AI (yes/no/unsure)* | Not sig  *p>0.05* |  | *Understanding of AI (yes/no/unsure)* | Not sig  *p>0.05* |  |
|  | *Confidence using AI technologies (4-point Likert)* | Not sig  *p>0.05* |  | *Confidence using AI technologies (4-point Likert)* | Not sig  *p>0.05* |  |
|  | *Confidence in AI terminology (4-point Likert)* | Significant  P=0.017  N=291 | V=0.158 (sml) | *Confidence in AI terminology (4-point Likert)* | Not sig  *p>0.05* |  |
|  | *Perception of adequacy of level of training (7-point Likert)* | Not sig  *p>0.05* |  | *Perception of adequacy of level of training (7-point Likert)* | Not sig  *p>0.05* |  |
|  | *Perception of having developed/learned some skill in AI (7-point Likert)* | Not sig  *p>0.05* |  | *Perception of having developed/learned some skill in AI (7-point Likert)* | Not sig  *p>0.05* |  |
|  | *Opinion on availability of training (7-point Likert)* | Not sig  *p>0.05* |  | *Opinion on availability of training (7-point Likert)* | Not sig  *p>0.05* |  |

***Supplementary Figure 1a and b: Coding of qualitative responses by profession***


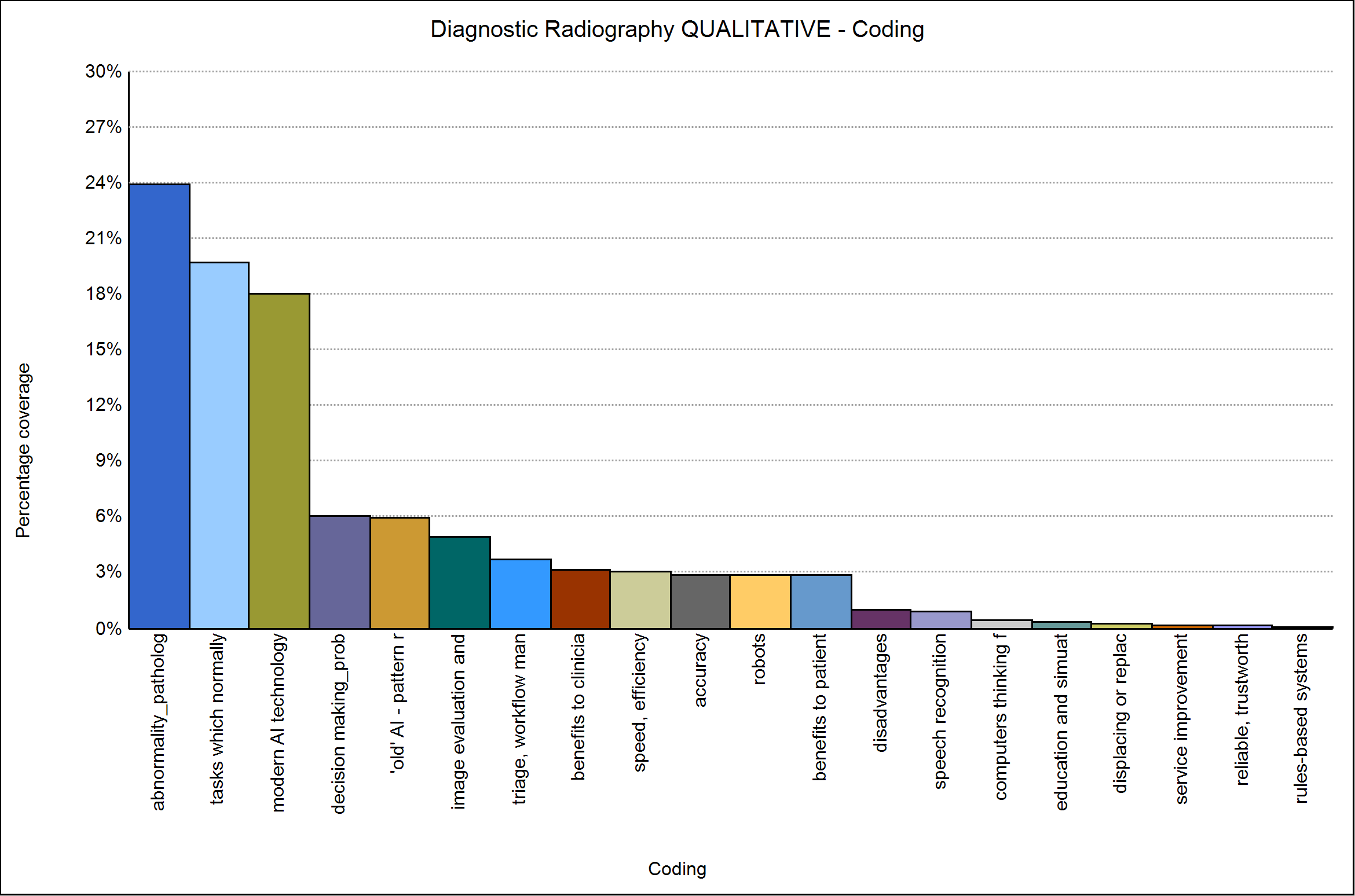


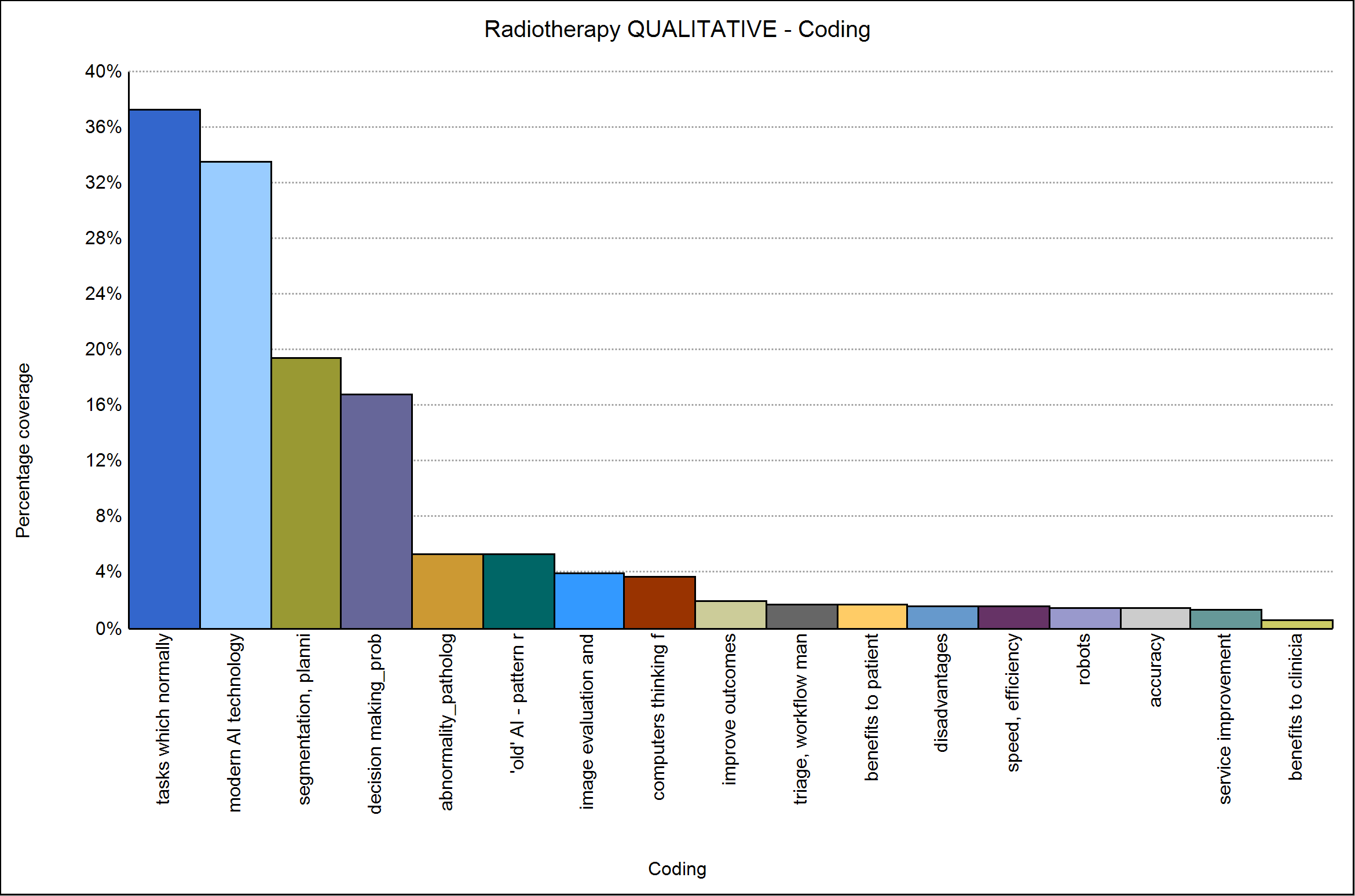


***Supplementary Figure 2: Thematic analysis of qualitative responses by profession***


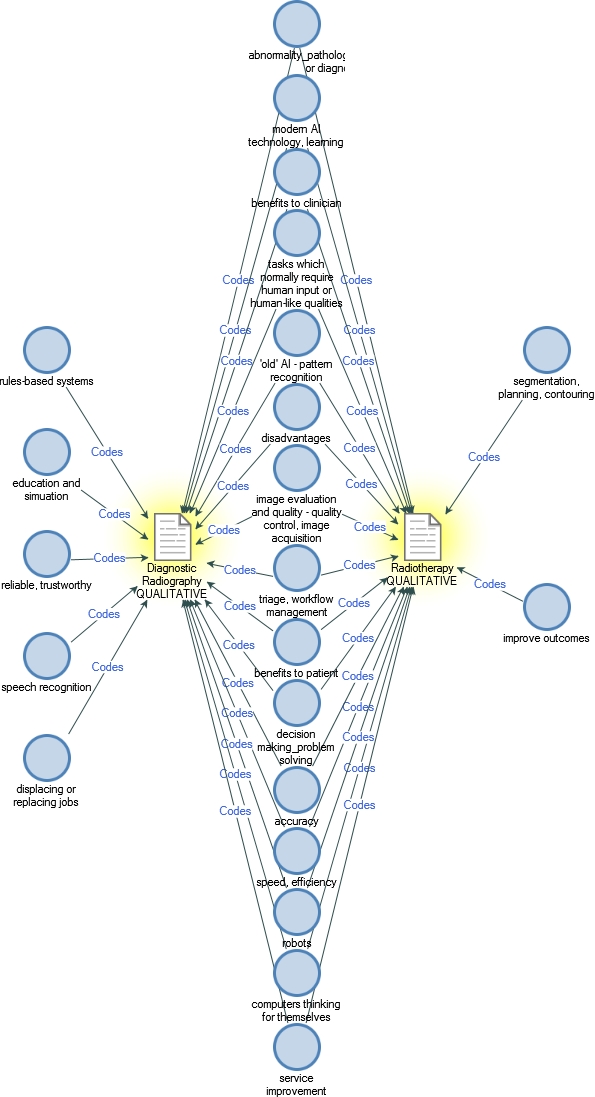

Supplement: Supplementary file 1 [file Data_Sheet_1.docx]
